# *TAMM41* is required for heart valve differentiation via regulation of PINK-PARK2 dependent mitophagy

**DOI:** 10.1038/s41418-019-0311-z

**Published:** 2019-03-01

**Authors:** Rui Meng Yang, Jiong Tao, Ming Zhan, Hao Yuan, Hai Hong Wang, Sai Juan Chen, Zhu Chen, Hugues de Thé, Jun Zhou, Ying Guo, Jun Zhu

**Affiliations:** 1 CNRS-LIA Hematology and Cancer, Sino-French Research Center for Life Sciences and Genomics, State Key Laboratory of Medical Genomics, Rui Jin Hospital, Shanghai Jiao Tong University School of Medicine, Shanghai, 200025 China; 2 Prenatal Diagnosis Center, Shanghai Jiao Tong University Affiliated First People’s Hospital, 650 Xin song jiang Road, Shanghai, 201620 China; 3 Department of Biliary-Pancreatic Surgery, Renji Hospital, School of Medicine, Shanghai Jiao Tong University, 160 Pujian Road, Shanghai, 200127 China; 4 Université de Paris 7/INSERM/CNRS UMR 944/7212, Equipe Labellisée No. 11 Ligue Nationale Contre le Cancer, Hôpital St. Louis, Paris, France; 5 Department of Cardiology, Shanghai Children’s Medical Center, School of Medicine, Shanghai Jiao Tong University, Shanghai, 200127 China

**Keywords:** Autophagy, Development

## Abstract

*TAMM41*, located within the congenital heart diseases (CHD) sensitive region of 3p25 deletion syndrome, is a mitochondrial membrane maintenance protein critical for yeast survival, but its function in higher vertebrates remains unknown. Via in vivo zebrafish model, we found that *tamm41* is highly expressed in the developing heart and deficiency of which led to heart valve abnormalities. Molecular mechanistic studies revealed that TAMM41 interacts and modulates the PINK1-PARK2 dependent mitophagy pathway, thereby implicating TAMM41 in heart valve development during zebrafish embryonic cardiogenesis. Furthermore, through screening of the congenital heart diseases (CHD) sensitive region of 3p25 deletion syndrome among 118 sporadic atrioventricular septal defect (AVSD) patients, we identified three cases carrying heterozygous pathogenic intronic variants of *TAMM41*. All three cases lacked normal full-length *TAMM41* transcripts, most likely due to specific expression of the mutant allele. Collectively, our studies highlight essential roles for TAMM41*-*dependent mitophagy in development of the heart and provide novel insights into the etiology of AVSD.

## Introduction

Congenital heart disease (CHD), a multifactorial disorder associated with both genetic and environmental factors, is one of the leading types of birth defect and can lead to fatal consequences [1, 2]. Atrioventricular septal defect (AVSD) is a subset of malformations affecting heart valve formation that comprises around 7.4% of all CHDs [3]. Understanding the etiology of CHD is challenging due to both the intrinsic complex biological patterns of cardiogenesis and the extrinsic diversified clinical manifestations [4–6]. Epidemiology studies have determined genetic factors as a primary contributor to CHD [2]. However, only around 20% of CHD cases can be attributed to known genetic factors, such as copy number variations or de novo mutations, with most others remaining unknown [7–9]. Chromosomal deletion syndromes, though with a low incidence, can provide important clues in identifying CHD candidate genes. Combining mutation analyses of potential candidate genes in sporadic AVSD cases could be a preferable and effective choice in precisely determining causative genes. The human distal 3p deletion syndrome is a rare contiguous gene deletion of chromosome 3p25-pter [10, 11]. Characteristic features of this syndrome include cognitive handicap, growth retardation, microencephaly, and facial dysmorphisms. CHD, especially AVSD, occurs in about one-third of these patients. Phenotype and genotype correlation studies have helped to narrow down candidate 3p25 AVSD target genes within a region between *TIMP4* and *ATP2B2*, but no conclusive results have been obtained [12–15].

When valve formation initiates, myocardial cells located in the atrioventricular canal (AVC) differentiate and gradually acquire distinct character from other myocardial cells for further instructing endocardial cells within the endocardial cushions to delaminate from the surface layer, which then undergo trans-differentiation to become mesenchymal cells [16, 17]. Interestingly, increasing evidences suggest that the changing dynamics of cellular components, including mitochondria, are important regulators of cell differentiation. Indeed, unlike the relatively static nature of mitochondria within adult heart tissues, it has been found that mitochondria in the embryonic heart are more dynamic and undergo constitutive fusion, fission, and mitophagy process. Developmental associated mitophagy was initially identified needed for the maturation of erythrocyte, which was then found also required for the differentiation of other cell types such as neurons and macrophages [18]. It has been reported that zebrafish lacking autophagy-related genes led to defective heart looping and aberrant heart valve development [19]. However, exactly how mitophagy is implicated in development of the heart valve remains unanswered.

*TAMM41*, located within 3p25, is an extrinsic component of the mitochondrial inner membrane. Previous studies in yeast suggest that Tam41 is required for cardiolipin synthesis and for maintaining the functional integrity of the Tim23 protein translocator complex [20, 21]. Regardless, an exact role for *TAMM41* in cardiac development within higher vertebrates has not yet been investigated. In this study, we found that *tamm41* deficiency caused abnormal heart development in zebrafish. Molecular mechanistic studies reveal that TAMM41 participates in regulating heart valve formation through mediating PINK1-dependent mitophagy. Further through a screening the 3p- CHD sensitive region among 118 unrelated AVSD cases, two heterozygous intronic variations in *TAMM41* were identified in three patients. Aberrant splicing events, accompanied by reduced levels of normal functional transcripts were found in all three cases. Allele-specific expression (ASE) of *TAMM41* has been proposed as reason for the penetrance of the inherited defect in affected children. Thus, for the first time, our results identify *TAMM41* as a candidate gene, which has an influential role in CHD.

## Results

### *Tamm41* is required for zebrafish heart valve development

**Fig. 1:**
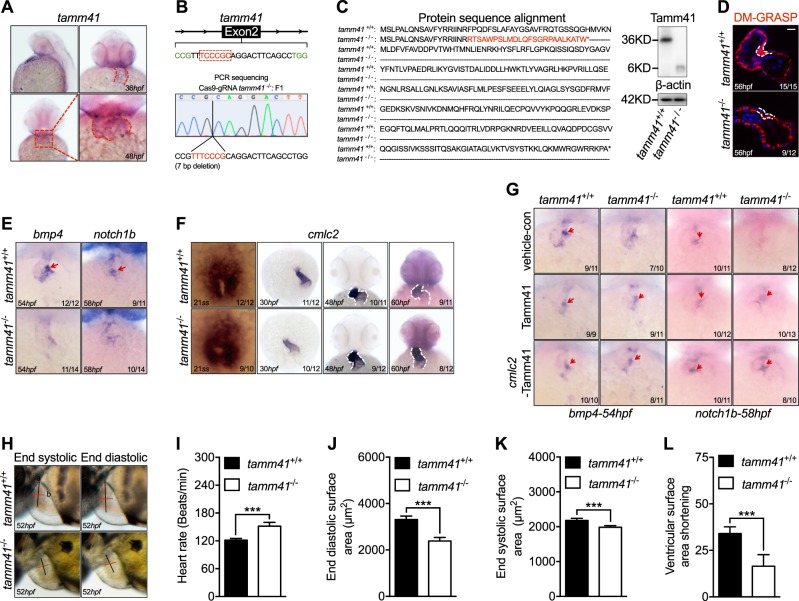
*Tamm41* is indispensable for zebrafish cardiogenesis. **a** WISH analysis of *tamm41* expression in zebrafish embryos at 36 hpf and 48 hpf. Red dotted lines delineate how *tamm41* expressed. **b** Schematic diagram of *tamm41* targeted crispr9 design, and transmittable F1 carrying 7 bp deletion in the exon2 of *tamm41* was generated. **c** The deletion caused a frame shift and generated a premature stop codon leading to truncated Tamm41 (6KD) generation without most of the functional domains. **d** Representative images show DR-GRASP staining of 56 hpf *tamm41*^+/+^ and *tamm41*^−/−^ hearts. While several layers of myocardium (upper panel outlined by white dashed line) form adjacent to the endocardial cushion in *tamm41*^+/+^ hearts, only a single layer of cardiomyocytes (lower panel outlined by white dashed line) is observed in *tamm41*^−/−^ hearts. Scale bar: 20 μm. **e** Representative images show the expression of heart valve formation markers *bmp4* and *notch1b* at the indicated times. While these markers are restrictively expressed in the AV canal region in *tamm41*^+/+^ embryos (upper panels, red arrows), they are weakly diffused throughout the whole hearts of *tamm41*^−/−^ embryos (lower panels). **f** Representative images of WISH assay of cardiomyocyte maturation marker *cmlc2* expression at the indicated times in *tamm41*^+/+^ and *tamm41*^−/−^ embryos. No obvious differences were detected. White dashed lines indicate the heart morphology outlined by *cmlc2* expression. **g** WISH analysis of *bmp4* and *notch1b* expression with *tamm41* or cmlc2-*tamm41* overexpression in *tamm41*^+/+^ and *tamm41*^−/−^ embryos. The abnormal *bmp4* and *notch1b* expression patterns in *tamm41*^−/−^ hearts are restored with either enforced *tamm41* or cmlc2-*tamm41* expression. Red arrows indicate normally restricted expression of *bmp4* or *notch1b* in the heart valve. **h**–**l** Optical Heartbeat analysis of cardiac function. Representative images showing ventricular at peak diastole and systole stage which are taken from movies of beating hearts (**h**). The heart rates (**i**), end-diastolic area (EDA) (**j**), and end systole area (ESA) (**k**) of *tamm41*^+/+^ and *tamm41*^−/−^ embryos (52 hpf, *n* = 10 for each group) are calculated by measuring the corresponding long (black line; a) and short (red line; b) axis (EDA or ESA = a/2 × b/2 × π). Fractional area changes of the ventricle (ventricular surface area shortening) (**l**) are measured by (EDA-ESA)/EDA × 100). Data are presented as mean ± SD. ***P < 0.001 (Student’s *t*-test)

To find out whether *TAMM41* was involved in heart development, we examined its function using zebrafish models, which have proved an excellent model for studying human cardiovascular disease [22]. *Tamm41* is conserved from yeast to higher vertebrates and mammals based on both synteny (Fig S1A) and protein sequence homology analyses. In zebrafish, the expression of *tamm41* is enriched in the developing heart from 36 hpf and became more apparent at later stages (Fig. 1a). To clarify the role of *tamm41*, a crispr/cas9 (Clustered Regularly Interspersed Short Palindromic Repeats) mediated *tamm41* knockout zebrafish line was generated (Fig. 1b). A 7 bp deletion was occurred in the second exon of *tamm41*, creating a premature stop codon, which resulted in a truncated Tamm41 protein lacking the majority of its functional domains (Fig. 1c). To better examine the heart valve formation, alcam staining (which stains all cardiomyocytes and endocardial cells at the AV boundary) was performed. In wild type hearts, cardiomyocyte cells adjacent to the AV canal were folded into 3–4 tiers, while in *tamm41*-deficient hearts, immature heart valves formed with only a single layer of cardiomyocytes (Fig. 1d). In zebrafish heart valve formation initiates with the gradual suppression of TGF-β signaling-related genes, such as *bmp4*, in the myocardium surrounding the AV canal from 48 hpf. However, we found that while *bmp4* expression remained weakly diffused throughout the myocardium in *tamm41* mutants at 58 hpf, the endocardial *notch1b* signal was also weaker than that of wild type embryos (Fig. 1e). The second heart field, which is important for heart valve development, was also normally developed as shown by *ltbp3* expression (Fig S1B). Moreover, expressions of genes for myocardial maturation (*amhc, vmhc,* and *cmlc2*) (Fig. 1f and S1C) and cardiac progenitor specification (*gata4, gata6* and *nkx2.7*) were undisturbed in *tamm41* mutants (Fig S1C). Heart valve abnormalities in *tamm41* mutants were rescued with *tamm41* mRNA overexpression, confirming a role for *tamm41* in the development of these heart valve abnormalities (Fig. 1g). To verify that the function of *tamm41* was specifically exerted in cardiomyocytes, overexpression of *tamm41* under the control of myosin light chain 7 was performed (*myl7*, a cardiomyocyte-specific promoter). Indeed, heart valve markers were restored with *myl7* promoter driven *tamm41* expression, confirming an autonomous function of *tamm41* in cardiomyocytes (Fig. 1g). *Tamm41* ablation also impaired heart function as shown by reduced end-diastolic surface area, end systolic area and ventricular surface area shortening at 52 hpf (Fig. 1h-l). In conclusion, these results suggest that *tamm41* is indispensable for heart development.

### *Tamm41-*deficient cardiomyocytes exhibit abnormal mitochondrial behavior

**Fig. 2:**
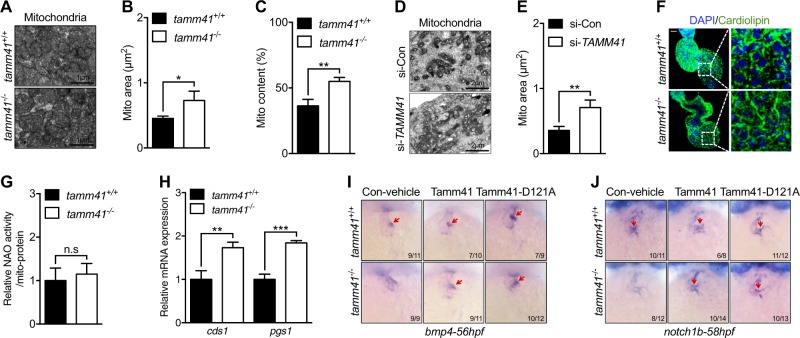
*Tamm41* deficiency leads to cardiomyocytes mitochondrial defects. **a**–**c** TEM images show enlarged mitochondria (lower panel) in the heart tissues of *tamm41*^−/−^ embryos compared with mitochondria (upper panel) in *tamm41*^+/+^ cardiomyocytes. **b** and **c** are quantitative data for mitochondrial areas and mitochondrial contents in *tamm41*^+/+^ and *tamm41*^−/−^ hearts based on TEM images respectively. **d**, **e** TEM analysis of human cardiomyocyte cell line (AC16) reveals enlarged mitochondria formation with *TAMM41* deficiency. Mitochondrial area quantitation is shown in (**e**). **f** Immunofluorescence analysis of cardiolipin revealed no obvious differences in fluorescence signals between *tamm41*^+/+^ and *tamm41*^−/−^ hearts. Right are the enlarged ones. Scale bar: 20 μm. **g** Relative CL level assessed by NAO in mitochondria isolated from 52hpf *tamm41*^+/+^ and *tamm41*^−/−^ embryos. **h** Elevated cardiolipin synthesis-related genes (*cds1* and *pgs1*) in *tamm41*^−/−^ embryos. **i**, **j** WISH assessment of valve-related markers with *tamm41*, *tamm41*-D121A mRNA overexpression. *Tamm41*-D121A, which has been suggested defective in cardiolipin synthesis, harbors the same effects in restoring the heart valves of *tamm41*^−/−^ embryos (third panel in **i** and **j**, red arrows) as with *tamm41* overexpression (second panel in **i** and **j,** red arrows). Black horizontal lines indicate mean ± SD. Means ± SD are shown for three independent experiments. n.s, not significant, *P < 0.05; **P < 0.01; ***P < 0.001 (Student’s *t*-test)

To study the abnormal heart development of *tamm41*-deficient embryos, the hearts of wild-type and *tamm41* mutants at 3*dpf* were analyzed using electron microscopy (EM). Wild type CMs exhibited highly organized thick and thin myofilaments clustered around many regular mitochondria (Fig. 2a–c). In contrast, larger and more elongated mitochondria were observed in *tamm41-*deficient zebrafish CMs (Fig. 2a–c). To validate this, we knocked down *TAMM41* by siRNA in AC16, a human cardiomyocyte cell line (Fig S2A). Consistently, enlarged hyperfused mitochondria were also a predominant feature of these *TAMM41* deficient AC16 cells (Fig. 2d, e). Tam41 has previously been reported to be required for the initial step in yeast cardiolipin biosynthesis [20]. However, the role for *TAMM41* in higher vertebrates remains unknown. In order to address this question, we first assessed cardiolipin levels. Surprisingly, WT and *tamm41-*depleted zebrafish embryonic hearts showed the same level of fluorescence intensity using cardiolipin-specific antibody staining (Fig. 2f). Consistent with this data, 10-N-nonyl-acridine orange staining (NAO, a cardiolipin-specific staining dye) [23] of isolated mitochondria from WT and *tamm41* mutants at 52 hpf, also revealed similar fluorescence intensities (Fig. 2g). It is worth noting that several other genes also related to cardiolipin biosynthesis, such as *cds1*, which are also reported as a cardiolipin synthesis enzyme in both the mitochondrial and endoplasmic reticulum, were found increased in *tamm41-*mutant embryos (Fig. 2h). These results imply that in multicellular organisms, the compromised synthesis of cardiolipin metabolic intermediates caused by mitochondrial *tamm41* deficiency might instead be compensated by the increased activity of other functionally similar proteins or indeed, supply from other organelles such as the endoplasmic reticulum (ER)-derived CDP-DAG. It has also been shown that the yeast Tam41-D220A mutation can abolish its enzymatic activity. In order to simulate this mutation, we mutated the corresponding conserved 121^st^ amino acid of zebrafish Tamm41 from aspartic acid to alanine (Tamm41-D121A) (Fig S2B) and performed rescue experiments in *tamm41* mutants. Overexpression of Tamm41-D121A in *tamm41* mutants achieved restoration of normal heart valve development, similar to Tamm41-WT (Fig. 2i, j). These observations suggest that cardiolipin biosynthesis is dispensable for the cardiac defects induced by *tamm41*-deficiency.

### Impaired mitophagy in the heart valve in *tamm41* deficient hearts

**Fig. 3:**
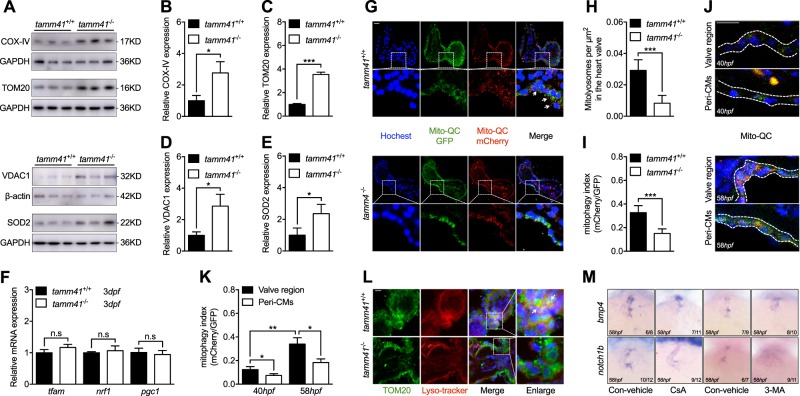
Disturbed mitophagy progression *tamm41* deficient embryos. **a**–**e** *Tamm41*^−/−^ embryos exhibit increased IMM (COX-IV), OMM (VDAC1, TOM20), and matrix (SOD2) protein levels revealed via western blot analysis. **f** Normally expressed mitochondrial biogenesis genes (*tfam*, *nrf1*, *pgc1*) in *tamm41*^−/−^ embryos revealed by qPCR. **g**–**i** The mito-QC was used to detect the lysosome-located mitochondria in *tamm41*^+/+^ and *tamm41*^−/−^ hearts. Reduced red fluorescence intensity in *tamm41*^−/−^ heart valve, which is not observed in the circulating red blood cells. For each group, ten hearts were examined. Scale bar: 20 μm. **j**, **k** mito-QC examination of mitophagy activities at 40 hpf and 58 hpf hearts. For each heart (*n* = 10), a ratio of mCherry to GFP mean fluorescence intensity was generated for the valve or peri-cardiomyocytes (Peri-CMs) region. Scale bar: 20 μm. **l** Representative images manifest the increased delivery of mitochondria (TOM20, green) to lysosome (lyso-tracker, red) in *tamm41*^+/+^ hearts (shown by white arrows), as compared with that in *tamm41*^−/−^ hearts. For each group, ten hearts were examined. Scale bar: 20 μm. **m** Reduced heart valve genes, including *bmp4* and *notch1b*, caused by either CsA or 3-MA addition. Black horizontal lines indicate mean ± SD. Means ± SD are shown for three independent experiments. n.s, not significant, *P < 0.05; **P < 0.01; ***P < 0.001 (Student’s *t*-test)

**Fig. 4:**
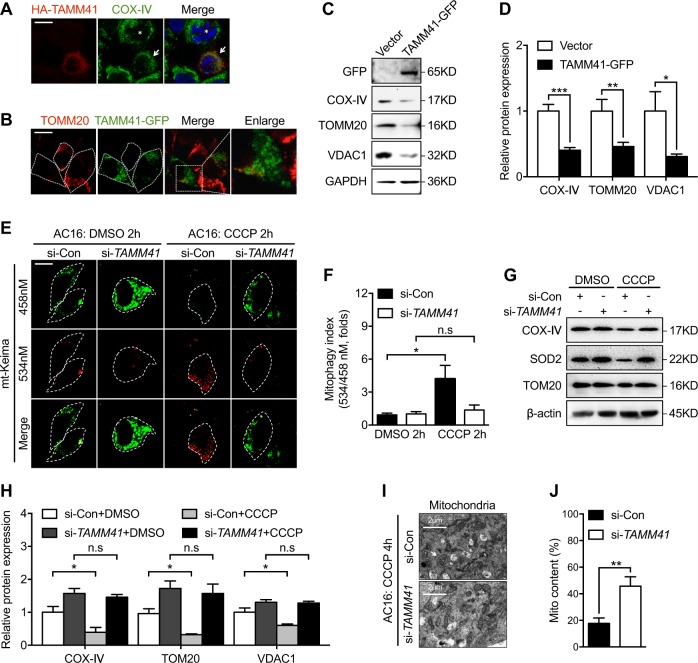
*Tamm41* regulates mitophagy progression in AC16 cells. **a** COS7 cells with HA-TAMM41 (red) overexpression manifest fragmented mitochondrial (COX-IV; green) appearance (white arrowhead) compared with cells without HA-TAMM41 transfection (asterisk). Scale bar: 5 μm. **b** Immunofluorescence assay shows diminished endogenous mitochondria (TOMM20, red) in TAMM41-GFP overexpressed 293T cells. Scale bar: 5 μm. **c**, **d** TAMM41 overexpression reduced mitochondrial protein levels in AC16 cells. **e**, **f** Mitophagy activity assessed using mt-Keima in si-control or si-*TAMM41* AC16 cells. Elevated levels of mitophagy were observed following CCCP treatment in control AC16 cells, which is not detected in *TAMM41* deficient ones. *n* = 50 cells calculated per group. Scale bar: 5 μm. **g**, **h** Western blot analysis reveal compromised CCCP-induced mitochondrial membrane protein elimination ability in *TAMM41*-deficient cells. **i**, **j** TEM (**i**) and mitochondrial contents calculation (**j**) show that while mitochondria engulfed by autophagosome (indicated by white arrow) and disappeared after CCCP treatment in si-control AC16 cells, enlarged mitochondria remain abundant (lower panel in **i**) in *TAMM41* deficient AC16 cells. Black horizontal lines indicate mean ± SD. Means ± SD are shown for three independent experiments. n.s, not significant, *P < 0.05; **P < 0.01; ***P < 0.001 (Student’s *t*-test)

Together with increased numbers of enlarged mitochondria in *tamm41* deficient heart tissues, several mitochondrial membrane-related proteins including, mitochondrial outer membrane protein (OMM) TOM20 and inner membrane protein (IMM) COXIV, were increased in *tamm41*-mutant zebrafish (Fig. 3a-e). This observation led us to make the assumption that *tamm41*-deficiency may perturb normal mitochondrial biosynthesis or degradation. However, the expression levels of several mitochondrial biogenesis related genes (*tfam*, *nrf1*, and *pgc1a)* remain undisturbed in *tamm41* mutant, suggesting a relative normal mitochondrial biosynthesis with *tamm41* deficiency (Fig. 3f). Mitophagy is a major degradation mechanism for the elimination of dysfunctional mitochondria through the autophagosome. The recently developed assays for measuring mitophagy include mt-Keima and mito-QC, both of which are pH-sensitive mitochondrial fluorescent probes that change color when they are transferred to an acidic environment like lysosome [24, 25]. Mito-QC consists of a mCherry-GFP fusion protein targeted to the outer mitochondrial membrane protein. The acidic environment of the lysosome would affect GFP fluorescence without influencing mCherry. We found both mitolyosome number and mCherry/GFP fluorescence intensity decreased in the *tamm41* mutant heart valves compared to WT controls (Fig. 3g-i). In fact, from 40 hpf to 58 hpf, elevated mitophagy activities were detected along with normal heart valves development (Fig. 3j, k). Consistently, we also observed that lysosome-located mitochondria were reduced in *tamm41*-depleted hearts (Fig. 3l). In order to verify the requirement of mitophagy for heart valve formation, Cyclosporine (CsA), an inhibitor of mitophagy, was then used [26–28]. As expected, mitophagy inhibition induced diffused *bmp4* and reduced *notch1b* expression in the valves (Fig. 3m). Similar results were obtained in the embryos treated with general autophagy inhibitor 3-methyladenine (3-MA) (Fig. 3m). Furthermore, through in vitro analysis, we observed that cells with higher TAMM41 expression often displayed fragmented or disappeared mitochondrial membrane protein (Fig. 4a-d). We also found that carbonylcyanide-3-chlorophenyl hydrazine (CCCP) induced mitophagy activity was largely reduced in *TAMM41-*deficient AC16 cells revealed by a decreased red signal of mt-Keima (Fig. 4e, h). Moreover, EM assay confirmed the failure of mitophagy dependent degradation of enlarged mitochondria in *TAMM41-*deficient AC16 cells even after CCCP treatment (Fig. 4i, j), reinforcing the hypothesis that *tamm41-*deficiency largely affects the clearance of mitochondria.

### *Tamm41* is required for the PARK2-PINK1-dependent mitophagy process

**Fig. 5:**
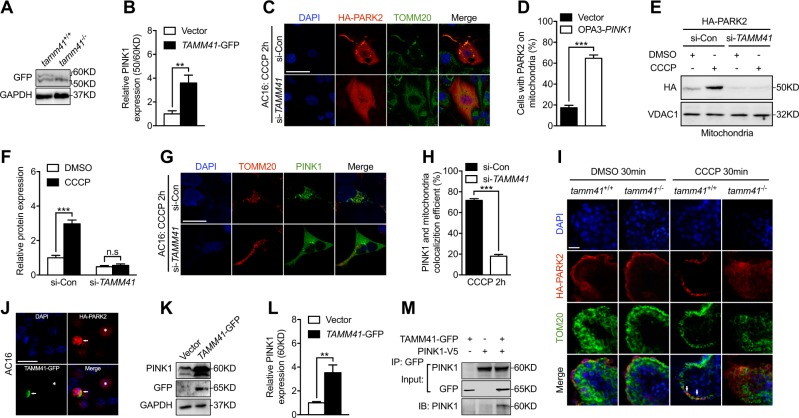
*Tamm41* is required for the PARK2-PINK1 mitophagy process. **a**, **b** Elevated unstabilized PINK1 (50KD) in *tamm41*^−/−^ embryos. **c**, **d** Representative images (*n* = 50 cells per group) show that *TAMM41* silencing affected CCCP induced HA-PARK2 (red) recruitment onto mitochondria (TOMM20, green) in AC16 cells. Scale bar: 20 μm. **e**, **f** Western blot assessment of mitochondrial-fractionated PARK2 levels in transfected AC16 cells. *TAMM41* deficiency inhibited PARK2 mitochondrial accumulation elicited by CCCP addition. **g**, **h** Representative images (*n* = 50 cells per group) show that reducing *TAMM41* expression impeded CCCP triggered PINK1-GFP mitochondrial stabilization (TOMM20, red). Scale bar: 20 μm. **i** Representative images (*n* = 10 per group) reveal that CCCP treatment induced HA-PARK2 (red) translocation onto mitochondria (TOM20, green) in *tamm41*^+/+^ hearts (white arrows), but failed to do so in *tamm41*^−/−^ hearts. Scale bar: 20 μm. **j** Enhanced HA-PARK2 recruitment onto mitochondria in TAMM41-GFP overexpressed AC16 cells (white arrowhead) compared with cell without TAMM41 overexpression (asterisk). Scale bar: 20 μm. **k**, **l** TAMM41-GFP overexpression promoted PINK1 stabilization (60KD) in the AC16 cell line by western blot. **m** Co-immunoprecipitation assay demonstrates the interaction between TAMM41-GFP and PINK1-V5. Black horizontal lines indicate mean ± SD. Means ± SD are shown for three independent experiments. n.s, not significant, **P < 0.01; ***P < 0.001 (Student’s *t*-test)

CCCP-induced mitochondrial inner membrane potential dissipation has been shown to impede PINK1 inner membrane transport and trigger its outer membrane stabilization, thus providing a critical signal for mitophagy progression [29]. Indeed, clearly increased unstable PINK1 was detected in *tamm41*-mutant zebrafish (Fig. 5a, b). AC16 cells with partial silencing of *TAMM41* also revealed significantly reduced translocation of PARK2 from the cytosol to damaged mitochondria following CCCP treatment as revealed by both IF and western blots analysis (Fig. 5c-f). Meanwhile, compromised PINK1 mitochondrial stabilization was also observed in *TAMM41* deficient cells (Fig. 5g, h). We also found that in WT hearts, CCCP addition triggered PARK2 mitochondrial translocation, which was otherwise disturbed in *tamm41*-mutant hearts (Fig. 5i). As the mitophagy inducing role of TAMM41 suggested above, TAMM41 overexpression could also accelerate PARK2 mitochondrial recruitment and increase PINK1 mitochondrial stabilization (Fig. 5j–l). To further elucidate the mechanism by which TAMM41 influenced PINK1 mitochondrial stabilization, an in vitro co-transfection coupled immunoprecipitation assay was performed. This experiment showed that the two proteins interacted directly (Fig. 5m), further support the notion that TAMM41 actively participates in regulating PINK1-dependent mitophagy.

**Fig. 6:**
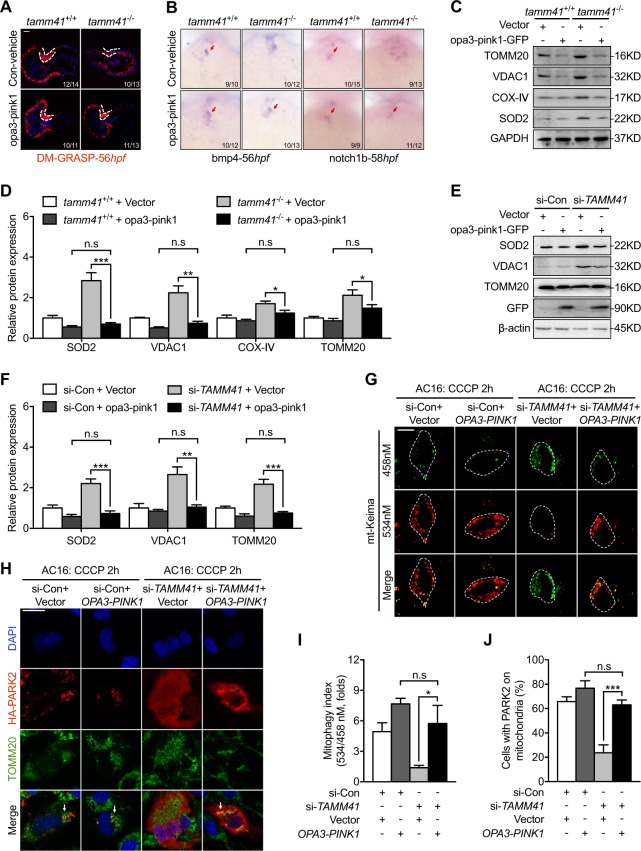
Mitochondria tethered Pink1 overexpression restores *tamm41* deficiency induced heart valve abnormalities. **a** Representative images show the recovering effect of *opa3-pink1* overexpression on 56 hpf *tamm41*^−/−^ heart valve via DR-GRASP staining. Scale bar: 20 μm. **b** *Opa3-pink1* injection at one-cell stage embryos restored heart valve markers, including *bmp4* and *notch1b*, in *tamm41*^−/−^ embryos. **c**, **d** Reduced mitochondrial membrane protein levels (fourth lane) in *tamm41*^−/−^ embryos with *Opa3-pink1* mRNA injection. **e**, **f** OPA3-PINK1 overexpression displayed reduced mitochondrial membrane proteins in si-*TAMM41* AC16 cells with CCCP addition (fourth panel). **g** Representative images (*n* = 50 per group) revealed mito-tethered PINK1 (OPA3-PINK1) overexpression restored CCCP triggered mitophagy activity in the *TAMM41* deficient AC16 cell line. Scale bar: 5 μm. **h** Representative images (*n* = 50 cells per group) show the enforced OPA3-PINK1 restored HA-PARK2 translocation onto mitochondria (indicated by white arrows) in the *TAMM41* deficient AC16 cell line with CCCP treatment. Scale bar: 20 μm. **i**, **j** (**i**) and (**j**) are quantitative data for (**g**) and (**h**) respectively. Black horizontal lines indicate mean ± SD. Means ± SD are shown for three independent experiments. n.s, not significant, *P < 0.05; **P < 0.01; ***P < 0.001 (Student’s *t*-test)

To verify that dysregulated Pink1 mitochondrial localization was a cause of heart defects in *tamm41*-deficient embryos, a mitochondrial-anchored pink1 construct was generated by replacing the first 110 amino acids of PINK1 with the outer mitochondrial membrane anchor from OPA3 (1-30) [30]. Importantly, overexpression of this mitochondrial tethered PINK1 restored heart valve abnormalities as revealed both Alcam staining (Fig. 6a) and WISH assessment of valve markers (Fig. 6b). In addition, mitochondrial proteins were also reduced in *tamm41*-mutant embryos with *opa3-pink1* mRNA injection (Fig. 6c, d). Then the effect of OPA3-PINK1 on the mitophagy defect caused by *TAMM41* deficiency was also examined. Defective CCCP induced mitophagy in *TAMM41-*depleted AC16 cells was recovered as shown by reduced mitochondrial membrane protein levels (Fig. 6e, f) and elevated mitochondria located lysosome with OPA3-PINK1 overexpression (Fig. 6g, i). PARK2 mitochondrial translocation was also returned normal (Fig. 6h, j). These results strongly suggest that defective mitophagy accounts for the defective valve development in *tamm41-*deficient hearts.

### Enforced mito-fission cannot restore heart development in *Tamm41*-deficient embryos

**Fig. 7:**
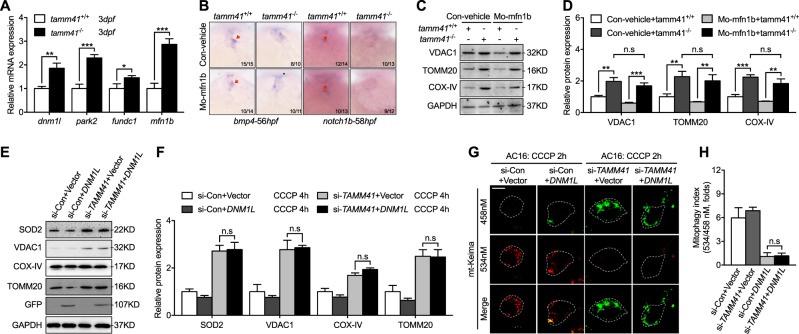
Enforced mitochondrial fission couldn’t restore heart valve malformation with *tamm41* mutant embryos. **a** qPCR analysis of mitophagy related gene expression (*dnm1l*, *park2*, *fundc1*, *mfn1b*) in *tamm41*^+/+^ and *tamm41*^−/−^ zebrafish embryos. **b** Representative images reveal the failure of MO-*mfn1b* injection to recover the abnormally expressed heart valve markers in *tamm41*^−/−^ embryos (lower panels). Red arrows indicate the normally expressed heart valve genes. **c**, **d** Representative images of three independent experiments revealed that elevated mitochondrial membrane proteins in *tamm41*^−/−^ embryos could not be restored with MO-*mfn1b* injection. **e**, **f** DNM1L-GFP overexpression failed to decrease the elevated mitochondrial membrane proteins in si-*TAMM41* AC16 cells with CCCP treatment (fourth panels). **g**, **h** Representative images (*n* = 50 cells) show that DNM1L-GFP overexpression is incapable of restoring defective mitophagy in *TAMM41* deficient AC16 cells with CCCP addition. Scale bar: 5 μm. Black horizontal lines indicate mean ± SD. Means ± SD are shown for three independent experiments. n.s, not significant, *P < 0.05; **P < 0.01; ***P < 0.001 (Student’s *t*-test)

Interestingly, our data showed that several regulators of mitochondrial dynamic activities including *mfn1b*, a gene encoding a mitochondrial outer membrane fusion protein, were increased in *tamm41*-mutant zebrafish (Fig. 7a). Mitochondrial membrane fusion and fission processes have been suggested to be intertwined with the mitophagy process in different contexts [31]. On the one hand, mitochondrial fission has been proposed as a preliminary step in the mitophagy process, whilst on the other hand, mitochondrial fusion could either serve as a protective feedback mechanism or a cause of defective mitophagy. To better understand the role of mitochondrial fusion in *tamm41*-deficient abnormal heart development, we examined the effects of mito-fusion inhibition by knocking down *mfn1b* with specific morpholinos. However, we observed no obvious restorative effects on the abnormal valve formed (Fig. 7b). Moreover, levels of mitochondrial membrane proteins remained elevated in *tamm41* mutant zebrafish with *mfn1b* morpholino injection (Fig. 7c, d). Hence, our results do not support a role for hyper mito-fusion in the pathogenesis of the *tamm41* mutant phenotype. In parallel, we examined the effect of enforced mito-fission on *TAMM41* deficient mitochondria. Dynamin-related protein (DRP)-1, a major mitochondrial fission promoting protein which is predominantly located in the cytosol and can promote mitochondrial fission when recruited to the outer mitochondrial membrane (OMM), was overexpressed in AC16 cells. Consistently, DRP-1 overexpression failed to reduce the elevated mitochondrial membrane proteins found in *TAMM41-*deficient cells (Fig. 7e, f). Immunofluorescence assays confirmed that the mitophagic process could not be restored (Fig. 7g, h). Thus, in *tamm41* deficient embryos, hyper-activation of mitochondrial fusion likely serves as a protective mechanism against defective mitophagy.

### Identification of pathogenic *TAMM41* intron variations in CHD patients

**Fig. 8:**
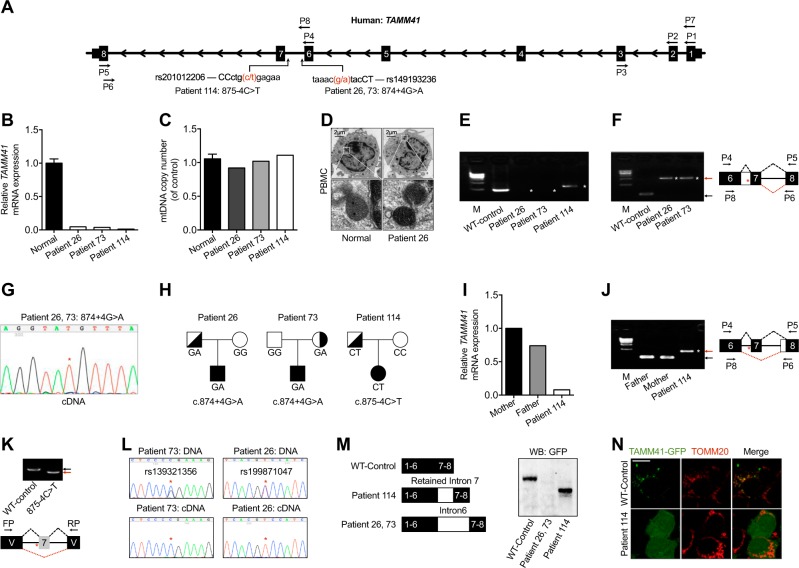
Variations of *TAMM41* identified in AVSD patients. **a** Schematic diagram of *TAMM41* transcript 005 (Ensemble ID: OTTHUMT00000339255) and the variations in Patient 26, 73, and 114 with their virtual positions in intron 6. **b** qPCR analysis of *TAMM41* from whole blood cell extracted RNA with primers P2 and P3 (shown in **a**). The results show significantly reduced *TAMM41* expression in the three affected children compared with six normal controls. **c** mtDNA copy number were evaluated by assessing the relative amounts of mitochondrial DNA and nuclear DNA (nDNA) of PBMCs from three patients and six healthy controls. MT-CO2 was used for as a marker for mtDNA and GAPDH for nDNA. **d** Representative TEM images showing mitochondrial morphology of PBMCs isolated from Patient 26 and one healthy control. Lower are enlarged ones showing mitochondria of the upper images. **e** Nest PCR with primers P1-P5 and P7-P6 (shown in **a**) failed to amplify full-length *TAMM41* in Patient 26 and 73, and revealed an abnormally longer product in Patient 114. **f** Left is the DNA agarose gel electrophoresis showing that whilst normally spliced transcripts including exon 6, 7, and 8 (indicated by black arrows) amplified with primers P4 and P5 in control samples, the intron 6 retained transcript was detected via nested PCR (P4-P5 and P8-P6, indicated in **a**) in Patient 26 and 73. Right is the schematic drawing showing the different splicing patterns between the normal control (black dashed lines) and the patient samples (red dashed lines). **g** Sequencing of the PCR products in Patient 26, 73 (Fig. 1f) revealed the transcription of which from the mutant allele (indicated by red asterisks). **h** Transmission pattern of the *TAMM41* variations identified in Patient 26, 73, and 114. **i**, **j** qPCR analysis indicating normal *TAMM41* expression in sample from the father of Patient 114. The amplification products using P4-P5 and P8-P6 of Patient114 (red arrow), and her parents (black arrow) are shown on the left. Right is the schematic drawing indicating the different splicing events. **k** The variation (875-4C>T) in the splicing minigene reporter caused inactivation of the nearby splicing acceptor site and led to exon 7-skipping transcript generation. Lower is the schematic drawing manifesting the splicing pattern. **l** Exclusive expression from one allele was found in Patient 26 (rs199871047) and Patient 73 (rs139321356). The difference between genomic genotype (double peaks) and cDNA genotype (single peak) in Patient 26 and 73 implied the monoallelic expression of *TAMM41*. Red asterisks indicate the SNP positions. **m** Western blot analysis reveals that while a truncated protein caused by a premature stop codon was generated by the aberrant transcript identified in Patient 114, no protein was detected from the intron 6 inserted construct (Patient 26 and 73). **n** Co-immunofluorescence of mitochondrial outer membrane marker TOMM20 (red) and TAMM41-GFP showed that the truncated TAMM41, generated from Patient 114’s aberrant transcript, failed to localize onto mitochondria. Scale bar: 5 μm. V, vector. M, marker. White asterisks in **e**, **f** and **j** indicate transcripts generated by nest PCR. Red asterisks in **f**, **g**, **j** and **k** indicate the variation site

To investigate the involvement of *TAMM41* in CHD development, 118 unrelated CHD patients of Han Chinese ancestry (diagnosed as simple AVSD or complex AVSD accompanied with more complex heart abnormalities) were enrolled (Supplemental Table 1) and all candidate genes located within the congenital heart diseases (CHD) sensitive region of 3p25 deletion syndrome were sequenced. Two types of heterozygous splice-region variants of *TAMM41* were identified in three patients: 875-4C>T in Patient114 (corresponds to rs201012206, with an allele frequency of 0.0003799, 55 alleles out of 144756 in the gnomAD database), 874+4G>A in Patient 26 and Patient 73 (corresponds to rs149193236, with an allele frequency in the gnomAD database of 0.0005294, 149 alleles out of 281432) (Fig. 8a). No combinations of any other gene mutations were identified in Patient 26 and Patient114, who were both diagnosed with AVSD. Patient 73 manifested more serious and complex clinical manifestations including, Single Ventricle (SV), Large Artery Dislocation (MGA), Pulmonary Atresia (PA) and AVSD. Aside from carrying the same variation of *TAMM41* (874+4G>A) as Patient 26, a missense variant of *SYN2* (rs2289706, c.1376A>G) was also identified in Patient 73. This may reflect the fact that combined mutations of susceptible genes may influence the severity of CHD. To verify whether additional CHD related pathogenic mutations were involved, whole exome sequencing of the three AVSD cases were performed. No common mutations or obviously CHD related mutations were identified (Supplemental Table 2). All three patients manifested greatly reduced *Tamm41* expression as shown by QPCR analysis from peripheral blood samples (Fig. 8b). However, the mitochondria of the patients’ PBMC seemed normal as both the mt-DNA copy number (Fig. 8c), which is an indication of mitophagy activity, and mitochondrial morphology examined by electron microscopy remained unchanged (Fig. 8d). This observation possibly suggested a tissue-specific role of *tamm41* in heart development.

Mutations near splicing sites could possibly lead to aberrant splicing events, a phenomenon which is estimated to account for around half of the alleles causing human disease [32, 33]. The full-length *TAMM41* transcript (containing exons 6, 7, and 8) was successfully amplified in control samples (Fig. 8e). However, as with Patient114, the 875-4C>T variation led to the inactivation of the adjacent 3’ acceptor site of intron 6. As a replacement, a novel site in intron 7 was used, which resulted in a partial intron 7 retained transcript (Fig. 8e). Meanwhile, intron 6 retained transcripts, exclusively transcribed from the mutant allele, were detected in Patient 26 and Patient 73, which was most likely caused by 874+4G>A variation induced 5’ splice donor inactivation (Fig. 8f, g). Most likely intron 6 retention produced a full-length transcript too long to be amplified by using common PCR, all aberrant transcript detected in the patient’s samples were obtained via nest PCR.

Genotyping of the corresponding variants in the patients confirmed a paternal or maternal transmission pattern (Fig. 8h). Although bearing the same mutated genotype, none of the parents manifested obvious heart disease and the father of Patient 114 exhibited a normal *TAMM41* splicing pattern (Fig. 8i, j). Similar genomic backgrounds leading to such distinct transcriptional manifestations are reminiscent of a biological phenomenon called allelic-specific expression (ASE). The contributions of ASE to phenotypic variation have been observed in a number of genetic diseases [34]. To confirm the pathogenic property of the mutant allele, the *TAMM41* exon 7 sequence (with flanking intronic sequences of either WT or mutant allele (875-4C>T) was cloned into a minigene reporter [35] and transfected into Hela cells. PCR amplification revealed inactivation of the intron 6 splicing acceptor site due to the 875-4C>T variation and resulted in the generation of an exclusive exon 7-skipped transcript (Fig. 8k). Indeed, previous investigations have implied that *TAMM41* was mono-allelically regulated in mouse fibroblasts (https://mae.hms.harvard.edu/dataanalysis.php). Accordingly, we then analyzed the ASE of *TAMM41* in our patients’ samples. Two heterozygous SNPs (rs139321356 and rs199871047) were found in the two 874+4G>A carriers. Intriguingly, sequencing analysis revealed that the cDNA, derived from whole blood cells, was indeed transcribed from only one allele of *TAMM41* (Fig. 8l), thus strongly indicating that ASE of *TAMM41* had occurred.

Finally, we set out to examine the potential protein products of the aberrant *TAMM41* transcripts found in the three patient samples. The full-length intron 7-containing transcript of Patient114 was cloned via nested PCR. Since full-length transcripts were undetectable in samples from Patient 26 and Patient 73, intron 6 was inserted into full-length wild type TAMM41, thus mimicking the aberrant transcription product (Fig. 8m). Interestingly, no protein expression was detected from the intron 6-insertion construct, possibly due to mRNA instability caused by retention of the intron (Fig. 8m). This finding correlated well with the observation that full-length TAMM41 transcripts were missing in Patient 26 and Patient 73. The partial intron 7-containing transcript from patient 114 introduced a premature stop codon, leading to the generation of a truncated TAMM41 protein (Fig. 8m), which failed to localize to the mitochondrial membrane (Fig. 8n). Collectively, our results show that *TAMM41* could be a candidate gene contributing to CHD.

## Discussion

Through genetic mapping of 3p-deletion patients, a critical missing region which confers susceptibility for the development of CHD has been determined [10, 11, 13]. However, confounding factors such as incomplete penetrance and long-range regulatory effects have made it difficult to precisely determine the causative genes [12, 14, 15]. Indeed, patients with large deletions of 3p but with normal phenotypes have also been reported [36]. In our study, within this critical region, we identified 3 out of 118 AVSD patients carrying pathogenic intronic *TAMM41* variations. All three patients manifested abnormally spliced and significantly reduced expression of normal *TAMM41* transcripts. Intriguingly, the biological father of Patient 114 had no obvious heart disease and *TAMM41* expression levels remained normal. Skewed X-inactivation and genomic imprinting have led to daughter/ maternal phenotype discrepancies in the past [37]. However, since few imprinting genes were identified, their contributions to the incomplete penetrance of CHD might be minor. As such, we favor the more widely reported phenomenon of monoallelic expression (MAE). MAE, an important regulatory mechanism for cellular heterogeneity and incomplete penetrance observed in multiple genetic diseases, occurs in specific cell types and shows temporal regulation [34]. A recent RNAseq study of heart tissue samples from CHD patients found that ASE events with significantly altered gene expression were important contributors to CHD development [38]. Within heart tissue, preferential expression of imprinted genes from the paternal allele has previously been reported. Hence, it would not be surprising if a similar preference for monoallelic gene expression (as demonstrated here in *TAMM41* mutant patients), also existed.

After the initial discovery of mitophagy in erythrocyte maturation, it has now been shown to participate in multiple biological processes, for instance, sperm motility and viability, midgut metamorphosis, and maintenance of stem cell pluripotency [39–41]. In adult hearts, mitophagy acts more as an inducible sensor against stress or injury [42]. However, investigations of the role of mitophagy during embryonic heart development using genetically modified mouse models have often been hampered by early lethality or functional compensation due to induction of ancillary or redundant pathways [42–44]. Indeed, a recent in vivo mitophagy assay using mito-QC found that active mitophagy did occur during mouse embryonic cardiomyocyte development [25] and furthermore, that autophagy inhibition induced heart valve abnormalities within zebrafish embryos [19]. The preferential use of a specific mitophagy pathway, for example, NIX activation of mitochondrial elimination in erythrocyte maturation, indicates that tissue or context-specific regulatory mechanisms may exist [45]. Thus our identification of the requirement of Tamm41-dependent mitophagy for heart valve development improves our understanding of the diversified roles of mitophagy played.

It has been found that mitochondria modulate cellular behavior through mechanisms like eradication ROS, metabolic remodeling and so on [46]. The so induced glycolysis by mitophagy is essential for the differentiation of neurons and macrophages. Likely, embryonic heart also prefers glycolysis as the main energy source. Moreover, in neonatal hearts, PARK2-PINK1-dependent mitophagy promotes mitochondrial metabolism transitions and myocardial maturation [47]. In our study, we found that in the heart valve formation regions, mitophagosomes were specifically increased and inhibition of mitophagy was sufficient to induce heart valve abnormalities in zebrafish embryos, as revealed by the defects in expression of *bmp4* and *notch1b* in the AV canal. So we postulate that activated mitophagy in myocardium may help to remodel a favorable metabolic environment to facilitate endocardial invasion or trigger specific pathways, for instance, Notch1 activation. In support of this, *TAMM41* expression has been reported to correlate with TGF-beta signaling [48]. Thus future studies are needed for illustrating the precise mechanisms inside.

## Materials and methods

### Human samples recruitment

A total of 118 unrelated AVSD patients, including 62 boys and 52 girls, aged 2–19 years-old, were recruited from Shanghai Children’s Medical Center. All subjects were of Chinese Han ethnicity. For each patient, medical history was taken, and physical examinations were performed. The final diagnosis was confirmed by echocardiography, cardiac magnetic resonance, or cardiac catheterization. Sixty-six patients were diagnosed as simple AVSD, with the remainder showing more complex heart abnormalities. Patients with Trisomy 21 were excluded. Detailed characterizations of the phenotypes are shown in Supplemental Table 3.

### Zebrafish maintenance

The maintenance, breeding, and staging of zebrafish were performed as previously described [49]. All animal works were strictly conducted following the guidelines of the Animal Care and Use Committee of Shanghai Jiao Tong University.

### Cells culture and reagents

Cells were cultured in humidified incubators at 37 °C in the presence of 5% CO_2_. The AC16 (ATCC) cell line was cultured in DMEM/F12 medium supplemented with 10% fetal bovine serum, 100 IU/ml penicillin and 100 μg/ml streptomycin (Gibco). 293T and COS7 were cultured in DMEM medium supplemented with 10% fetal bovine serum, 100 IU/ml penicillin and 100 μg/ml streptomycin (Gibco). Three siRNA of *tamm41* were designed and synthesized (GenePharma) and the one with the highest efficiency (5’-GCUGGCUGGAGAUAGAUAATT-3’) was used for the later experiment. siRNA was transfected using lipofectamine 2000 as instructed.

### Isolation of PBMC

PBMC were isolated from Patient 26 and a healthy control of the same age using BD Vacutainer® CPT™ Mononuclear Cell Preparation Tube according to manufacturer’s protocol. After washing twice, the deposited cells were subjected to electron microscopy analysis.

### Targeted next-generation sequencing and sequence data analysis

Seven genes in 3p25 CHD candidate region, including *HR1*, A*TG7*, V*GLL4*, *Corf31*, *SYN2*, *TIMP4*, and *CRELD1*, were selected for targeted sequencing. A multiplex-PCR based method, as described previously was employed to capture the targeted sequences [50]. The enriched amplification products were then size-separated, and the products of length 200–400 bp were recovered and subjected to DNA sequencing on MiSeq sequencer (Illumina) using Miseq reagent kit v2. The sequencing reads were separated for each sample by running CASAVA (Illumina Inc, San Diego, CA, USA) and the targeted reads were aligned to the human reference genome (hg19) using the Burrows–Wheeler Aligner (BWA). The SNVs in the targeted region were identified using the Genome Analysis Toolkit and Varscan programs [51], and the Annovar program was used for SNV annotation [52].

### Whole exome sequencing

Whole exome sequencing and variant filtering were performed using an Agilent SureSelect V6 enrichment capture kit (Agilent Technologies) with sequencing on the Illumina HiSeq x10 platform. Sequence reads were mapped to the human reference genome assembly (NCBI build 37/hg19) using BWA alignment with maximal exact matched algorithm [53]. Variant discovery and genotype calling of single nucleotide variants (SNVs), insertions and deletions were performed on all individuals using Sentieon DNA pipeline. To identify other potential pathogenic variants, the ones with snp142 Common record were removed. Then the minor allele frequency (MAF) of all detected variants was consulted across the databases of the ExAC, 1000 Genomes and ESP6500, and the ones with PopFreqMax > = 0.01 were filtered. The pathogenic variants were also evaluated using different in silico predictive algorithms like SIFT, Polyphen v2.

### Zebrafish husbandry and *Tamm41* knockout zebrafish generation

The Tg(*cmlc2*: GFP) line were used as previously described [54]. For crisp9 mediated *Tamm41* knockout zebrafish generation, guide RNA (gRNA) targeting exon2 of *Tamm41* was designed using an online tool ZiFiT Targeter software (http://zifit.partners.org/ZiFiT), which was synthesized by cloning the annealed oligonucleotides into the sgRNA expression vector as previously described. [55] The injected F0 founder embryos were raised to adulthood and then outcrossed with wild type zebrafish. F1 embryos carrying potential indel mutations were raised to adulthood. Then PCR amplification and sequencing were carried on genomic DNA isolated from tail clips of F1 fish to identify mutants.

### Plasmids cloning

The minigene splicing reporter was constructed as previously reported. [35] Generally, exon 6, intron 6, exon 7 of *Tamm41* with flanking genomic sequences were inserted into the vector. Vectors carrying mutant alleles were generated by a PCR-mediated mutagenesis strategy. PINK1-V5 (addgene#13320) and mt-Keima (addgene#56018) was obtained from Addgene. LC3 in vector FUW mCherry-GFP-LC3 (addgene, #110060) was replaced by mitochondrial targeting sequence of the outer mitochondrial membrane protein FIS1 for generating mito-QC. The so got mCherry-GFP-FIS1 was PCR amplified and cloned into pCS2 vector for in vitro transcription. Opa3-pink1 was cloned as previously described [30]. Zebrafish *tamm41*, *pink1*, *park2*, *dnm1l* were amplified from zebrafish cDNA. *Tamm41*-D121A was generated by PCR-mediated mutagenesis strategy from wild type zebrafish *tamm41*. Human *TAMM41* CDS were amplified from AC16 cDNA. For mRNA overexpression experiments, above suggested plasmids were then transcribed in vitro and 2 nl were injected at one-cell stage embryos. Tol2-plasmid was cloned by insertion of zebrafish *tamm41* CDS under *myl7* promoter (1.6k) as previously reported [54]. Transgenes were transiently expressed by co-injecting 20 pg of Tol2-plasmid as described above and 80 pg of Tol2 transpose mRNA at one-cell stage.

### Morpholinos and mRNA synthesis for microinjection

Zebrafish *tamm41* (5’-TGCAGAGCTGGAAGACTCATTTCTG-3’) and *mfn1b* (5’-TCTAACTGCTCCATTTCCACACTGT-3’) morpholino oligonucleotides targeting the transcriptional initiation ATG of *tamm41* and *mfn1b* were designed and purchased from Gene Tools. mMESSAGE mMACHINE Kit (Ambion) was used for mRNA transcription. Microinjection of morpholino oligonucleotides or mRNA was performed at the one-cell stage. All injections were performed with a Harvard Apparatus micro-injector.

### Heart function assessment

Cardiac function of sibling WT control and *tamm41* mutants were quantitatively assayed by the Optical Heartbeat analysis under an Olympus IX71 microscope with HC Image software.

### WISH; immunostaining of zebrafish hearts

Antisense digoxigenin-labeled RNA probes for *nkx2.7* [56]*, hand2* [57]*, gata6, gata4, ltbp3* [58], *cmlc2, vmhc, amhc* [59]*, bmp4, notch1b* [60], *tamm41* were transcribed in vitro using linearized constructs with T3 or T7 polymerase (Ambion) with the DIG-RNA Labeling Kit (Roche). Whole-mount mRNA in situ hybridization (WISH) was performed as described previously [61]. For assessing mito-QC activity in zebrafish, 40 hpf, 50 hpf and 52 hpf embryonic hearts were dissected and fixed one hour in 4% paraformaldehyde. After PBS washing twice, Hoechst 33342 (Invitrogen) were also added for visualizing cell nuclear confocal microscopy. For immunofluorescence assays, dissected hearts after fixing were incubated with primary antibodies overnight after blocking and permeabilization. Secondary antibodies were treated for two hours before the examination. For live cardiomyocyte confocal analysis, dissected hearts were incubated in Wittenberg isolation medium with 5 mg/ml BSA, 1.25 mg/ml taurine and 150 μM CaCl_2_ at room temperature. During CCCP (20 μM; 30 min) treatments, lysotracker-Green (200 nM; Life Technology), and nuclear hoechst 33342 were added. Antibodies used for zebrafish heart immunofluorescence assays were as follows: rabbit anti-TOMM20 antibody (Santa Cruz), human-anti-cardiolipin antibody (ImmunoVisi; on Springdale, AR, USA). Secondary antibodies (Invitrogen) used were: Alexa Fluor 488 goat anti-rabbit, Alexa Fluor 594 goat anti-rabbit, Alexa Fluor 488 goat anti-mouse, Alexa Fluor 488 goat anti-human.

### 10-N-nonyl-acridine orange (NAO) staining

For in vivo NAO staining, 26 hpf zebrafish embryos were subjected to NAO (2 μg/ml) for 30 min. After washing three times with PBS, embryos were then subjected to fluorescence microscopy examination. Mitochondrial isolated from 48 hpf zebrafish embryos were fixed in fixative buffer (0.25 M sucrose, 10 mM Tris-HCl, and 1% formaldehyde) for 15 min and stained with NAO (100 nM) for 20 min, followed by washing with PBS and measuring fluorescence at 485/530 nm.

### Western blot and coimmunoprecipitation

For Western blot, embryos at the indicated developmental stages were deyolked and homogenized in lysis buffer (20 mM Tris-HCl, pH 7.4, 150 mM NaCl, 5 mM EDTA, 10% glycerol, and 0.1% Triton X-100) as previously reported [62]. Proteins from cell lines in lysis buffer (20 mM Tris, pH 7.4, 137 mM NaCl, 2 mM EDTA, 10% glycerol, 1% NP-40 (NEW 728601), and protease inhibitor cocktail (Roche, 04693132001) were stored on ice for 40 min as previously described. For immunoprecipitation, the cells were lysed for 40 min on ice in lysis buffer containing protease inhibitors (Roche Applied Science, 04693132001). Cell lysates were incubated with the indicated antibodies and protein A/G-agarose beads (Invitrogen) overnight at 4 °C. The beads were extensively washed with lysis buffer and then dissolved in sample buffer containing 1% SDS for 5 min at 95 °C for western blot analysis. Zebrafish or cell line mitochondria were isolated using Tissue Mitochondrial Isolation Kit (beyotime) or Cell Mitochondrial Isolation Kit (beyotime), respectively, as instructed. The antibodies used for western blot were as follows: rabbit anti-TOM20 (Santa Cruz), mosue anti-GAPDH (Sigma), rabbit anti-COXIV (cell signaling), rabbit-anti-actin (Sigma), rabbit anti-TAMM41 (Sigma), rabbit-anti-PINK1 (cell signaling), rabbit-anti-SOD2 (cell signaling), mouse anti-GFP (Invitrogen), mouse anti-HA (Sigma).

### Cell immunofluorescence assay

The cells were grown to 60% confluence on coverslips. After each indicated treatment, cells were then fixed with freshly prepared 3.7% formaldehyde at 37 °C for 15 min, followed by washing 3 times with PBS. Samples were then incubated in blocking buffer (1X PBS/5% normal serum/0.3% Triton™ X-100) at room temperature for an hour, after which primary antibodies were added and incubated at 4 °C overnight, followed by washing 4 or 5 times with 0.05% Triton X-100 and staining with the secondary antibody for 1 h at room temperature. The antibodies used for cell IF analysis were as follows: rabbit anti-TOM20 (Santa Cruz), rabbit anti-COXIV (cell signaling), rabbit-anti-PINK1 (cell signaling), mouse anti-HA (Sigma).

### The mtDNA copy number quantification

mtDNA copy number were evaluated by assessing the relative amounts of mitochondrial DNA (mtDNA) and nuclear DNA (nDNA.). MT-CO2 was used for as a marker for mtDNA and GAPDH was used for nDNA. Primers are shown in Supplemental Table 4. For mRNA quantification, RNA was prepared using Trizol reagent (Invitrogen, H10522) and then subjected to complementary DNA synthesis using a cDNA synthesis kit (ABI). Real-time PCR was performed using a Fast Start Universal SYBR® Green Master (Rox) (Roche Applied Science, 13800300) probe and thermal cycler (Mastercycler; Eppendorf, 22331 Hamburg, Germany). Primers are summarized in Supplemental Table 4.

### Electron microscopy analysis

For electron microscopy, isolated PBMC or treated human cardiomyocyteAC16 cells were fixed with 2.5% glutaraldehyde for 1 h, followed by postfixation in 1% osmium tetroxide. Subsequently, the cells were dehydrated with increasing concentrations of ethanol, and embedded with epoxy resin. AC16 ultrastructures were taken using a transmission electron microscope (Hitachi, H-7650, Japan). For zebrafish heart electron microscopy analysis, the same procedures were performed on whole zebrafish embryos and ultra-thin sections of heart tissue were then examined using electron microscope (Hitachi, H-7650, Japan).

### Imaging

Zebrafish immunofluorescence staining images were taken using an Olympus FV1000 scanning confocal microscope. The dissected embryo hearts were mounted in 1% low-melt agarose. The confocal images were captured with an UPLSAPO 40×. Immunostained cell images were collected using a Leika with an UPLSAPO 60X objective.

### Image analysis

Mitochondrial area and content (percentage of mitochondrial area compared to the whole-cell area) were quantified using ImageJ. For all analysis, images in each group were obtained by uniform random sampling and processed by identical protocols. mito-QC labeled images were pre-processed by noise filtering and auto-thresholding, after which the mitolysosomes were typically identified on the basis of the thresholded red signal not overlapping with green. The ratio of mean fluorescence intensity (mCherry/GFP) was calculated as a value of mitophagy under laser scanning confocal microscopy. For each heart, three peri-CMs regions of the similar size with the corresponding valve region were randomly chosen. Similarly, as with mt-Keima, mitochondria in the lysosomes were identified by a high ratio of 543/458 nm. Parkin aggregation was counted as the number of cells with parkin translocation to mitochondria compared to all the cells.

### Study approval

Studies on human samples were approved by the Association of Medical Ethics of Shanghai Children’s Medical Centre. Fully informed consents were obtained from all parents or guardians. The zebrafish maintenance and study protocols were approved by the Institutional Review Board of the Institute of Health Sciences, Shanghai Institutes of Biological Sciences, Chinese Academy of Sciences (Shanghai, China).

### Statistics

Data were presented as mean ± standard deviation (SD). Group comparisons of normally distributed data were performed with unpaired Student’s *t*-test. SPSS 17.0 software (IBM, Chicago, IL, USA) was used for all statistical analysis. Values of *P* < 0.05 were considered statistically significant.

## Supplementary information

Supplemental Material

Supplemental table 1

Supplemental table 2

Supplemental table 3

Supplemental table 4
