# Supplementary material for: TAMM41 is required for heart valve differentiation via regulation of PINK-PARK2 dependent mitophagy
Source: Cell Death Differ. 2019 Mar 1;26(11):2430–46. doi: 10.1038/s41418-019-0311-z (PMC6888875; doi:10.1038/s41418-019-0311-z)
Supplement: Supplementary file 1 — Supplemental Material [file 41418_2019_311_MOESM1_ESM.pdf]

Supplementary material

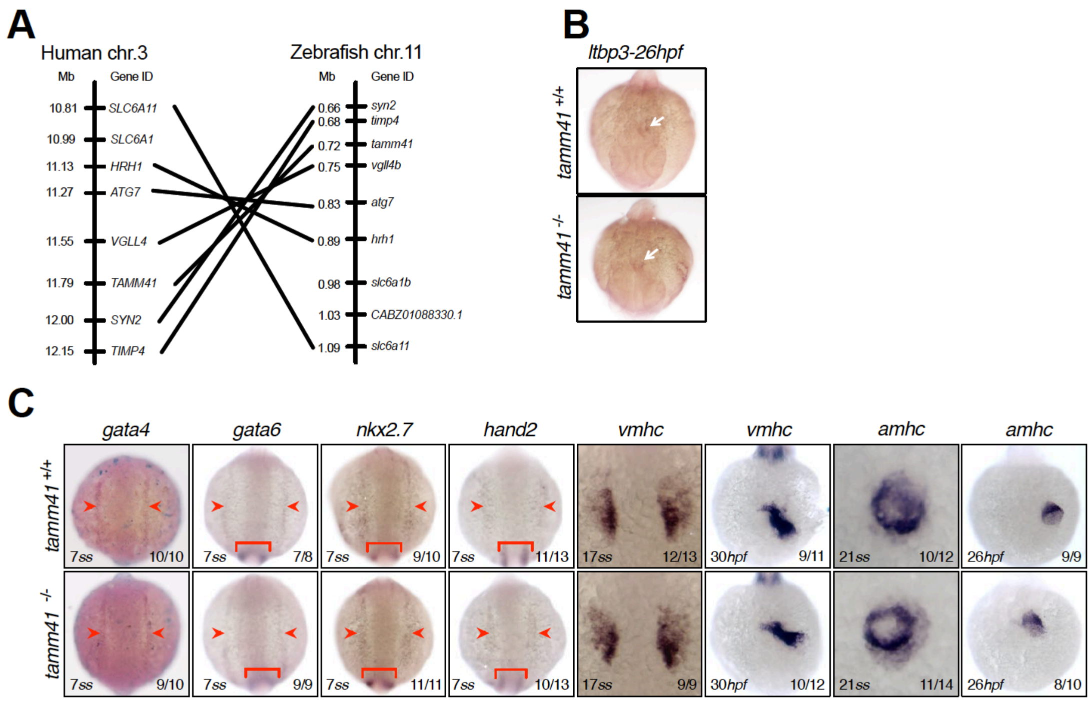

Fig S1. *Tamm41* is a conserved gene required for zebrafish heart development.

(A) Synteny analysis between zebrafish *tamm41* and human *TAMM41* loci.

(B) Expression of second heart field marker *ltbp3* (white arrows) stays unchanged at 26*hpf*.

(C) Representative images of WISH assay of heart progenitor markers *gata4*, *gata6*, *nkx2.7, hand2* and cardiomyocyte maturation markers *amhc, vmhc* at the indicated times in *tamm41*$^{+/+}$ and *tamm41*$^{-/-}$ embryos. No obvious differences were detected.

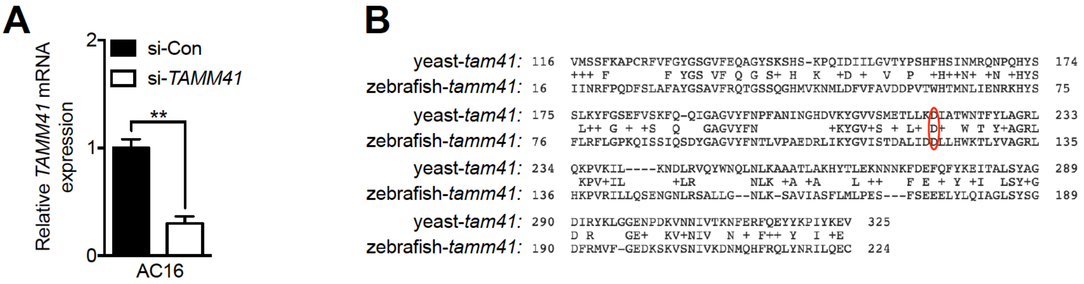

Fig S2. ***Tamm41*** **deficiency triggers mitochondrial abnormalities.**

(A) The knockdown efficiency of *Tamm41* si-RNA was verified by qPCR.

(B) The conserved zebrafish Tamm41-121D with respect to yeast Tam41-220D as indicated by red circle.

Black horizontal lines indicate mean ± SD. Means ± SD are shown for three independent experiments. ***P* <0.01 (Student's t-test).
