# Supplementary material for: TAMM41 is required for heart valve differentiation via regulation of PINK-PARK2 dependent mitophagy
Source: Cell Death Differ. 2019 Mar 1;26(11):2430–46. doi: 10.1038/s41418-019-0311-z (PMC6888875; doi:10.1038/s41418-019-0311-z)
Supplement: Supplementary file 2 — Supplemental table 1 [file 41418_2019_311_MOESM2_ESM.pdf]

Table S1: Phenotype of 118 Enrolled CHD Patients

**Phenotype**	**Number of Patients (N=118)**
AVSD	66
DORV+AVSD	25
SV+AVSD	17
TOF+AVSD	5
PA+AVSD	2
D-TGA+AVSD	3

AVSD: Atrio-Ventricular Septal Defect; DORV: Double Outlet Right Ventricle; SV: Single Ventricle; TOF: Tetralogy of Fallot; PA: Pulmonary Atresia; D-TGA: Complete Transposition of the Great Arteries.
