# Supplementary material for: TAMM41 is required for heart valve differentiation via regulation of PINK-PARK2 dependent mitophagy
Source: Cell Death Differ. 2019 Mar 1;26(11):2430–46. doi: 10.1038/s41418-019-0311-z (PMC6888875; doi:10.1038/s41418-019-0311-z)
Supplement: Supplementary file 3 — Supplemental table 2 [file 41418_2019_311_MOESM3_ESM.pdf]

Supplemental Table 2

Patient 26								
**Chromosome**	**Position**	**REF**	**ALT**	**REF-depth**	**ALT-depth**	**Total-depth**	**Gene**	**ExonicFunc.refGene**
chr1	1847930	G	C	188	125	313	CALML6	nonsynonymous SNV
chr1	8482818	A	T	14	27	41	RERE	nonsynonymous SNV
chr1	17257009	A	G	343	115	458	CROCC	nonsynonymous SNV
chr1	54474676	C	G	182	146	328	LDLRAD1	nonsynonymous SNV
chr1	55130855	T	G	71	71	142	MROH7	nonsynonymous SNV
chr1	75114940	G	A	32	50	82	ERICH3	nonsynonymous SNV
chr1	101343357	C	G	63	31	94	EXTL2	nonsynonymous SNV
chr1	144930603	T	C	104	28	132	PDE4DIP	nonsynonymous SNV
chr1	183602246	G	C	113	92	205	ARPC5	nonsynonymous SNV
chr1	200776529	C	A	31	31	62	CAMSAP2	nonsynonymous SNV
chr1	225190558	T	C	50	32	82	DNAH14	nonsynonymous SNV
chr2	10747427	G	C	58	35	93	NOL10	nonsynonymous SNV
chr2	103236521	A	C	295	266	561	SLC9A2	nonsynonymous SNV
chr2	113737699	G	A	60	48	108	IL36G	nonsynonymous SNV
chr2	198593217	C	T	80	62	142	BOLL	nonsynonymous SNV
chr2	207632111	C	G	71	56	127	FASTKD2	nonsynonymous SNV
chr2	220348315	C	T	18	29	47	SPEG	nonsynonymous SNV
chr2	231115740	C	T	13	21	34	SP140	stopgain
chr3	10302071	A	G	104	131	235	TATDN2	nonsynonymous SNV
chr3	49337979	G	A	59	44	103	USP4	nonsynonymous SNV

chr3	63898380	CAG	C	24	31	55	ATXN7	frameshift deletion
chr3	63898383	CAGCAGCA	C	27	32	59	ATXN7	frameshift deletion
chr3	78685145	T	G	57	47	104	ROBO1	nonsynonymous SNV
chr3	100962830	C	T	55	38	93	IMPG2	nonsynonymous SNV
chr3	184009226	A	G	145	176	321	ECE2	nonsynonymous SNV
chr4	8869951	G	A	41	48	89	HMX1	nonsynonymous SNV
chr4	75248500	T	A	30	33	63	EREG	stopgain
chr4	100063868	C	T	19	28	47	ADH4	nonsynonymous SNV
chr4	187540578	G	A	53	33	86	FAT1	nonsynonymous SNV
chr5	68412391	G	A	34	25	59	SLC30A5	nonsynonymous SNV
chr5	72419763	C	T	128	106	234	TMEM171	nonsynonymous SNV
chr5	115351065	T	TATATATATA TATATGGAA CTAAGACTA	60	8	68	LVRN	stopgain
chr5	125939860	A	G	41	24	65	PHAX	nonsynonymous SNV
chr5	134076989	G	A	150	131	281	CAMLG	nonsynonymous SNV
chr5	140221401	C	G	77	69	146	PCDHA8	nonsynonymous SNV
chr5	148407452	C	T	215	161	376	SH3TC2	nonsynonymous SNV
chr6	10529218	A	G	30	52	82	GCNT2	nonsynonymous SNV
chr6	25967061	G	A	219	208	427	TRIM38	nonsynonymous SNV
chr6	36653548	C	G	130	104	234	CDKN1A	nonsynonymous SNV
chr6	72006080	A	G	44	24	68	OGFRL1	nonsynonymous SNV
chr6	149887557	G	A	50	42	92	GINM1	nonsynonymous SNV
chr7	6227313	C	T	35	31	66	CYTH3	nonsynonymous SNV

chr7	11075368	A	T	104	87	191	PHF14	nonsynonymous SNV
chr7	12391283	C	G	41	28	69	VWDE	nonsynonymous SNV
chr7	63538921	A	G	64	36	100	ZNF727	nonsynonymous SNV
chr7	73790587	C	T	193	231	424	CLIP2	nonsynonymous SNV
chr7	100179690	C	T	35	40	75	LRCH4	nonsynonymous SNV
chr7	141352630	C	A	17	22	39	AGK	nonsynonymous SNV
chr7	143884953	T	C	11	14	25	ARHGEF35	nonsynonymous SNV
chr7	149463017	C	G	128	140	268	ZNF467	nonsynonymous SNV
chr7	149522451	T	C	107	98	205	SSPO	nonsynonymous SNV
chr8	3265726	G	A	41	42	83	CSMD1	nonsynonymous SNV
chr8	21769976	C	T	74	61	135	DOK2	nonsynonymous SNV
chr8	22436516	G	C	32	27	59	PDLIM2	nonsynonymous SNV
chr8	24298653	T	C	65	54	119	ADAM7	nonsynonymous SNV
chr8	30585143	G	C	79	62	141	GSR	nonsynonymous SNV
chr8	133637681	C	G	48	60	108	LRRC6	nonsynonymous SNV
chr8	145168722	G	A	71	59	130	WDR97	nonsynonymous SNV
chr9	32543694	G	A	56	54	110	TOPORS	nonsynonymous SNV
chr9	84607168	C	T	180	200	380	SPATA31D1	nonsynonymous SNV
chr9	96438025	C	T	190	161	351	PHF2	nonsynonymous SNV
chr9	101530527	T	C	123	168	291	ANKS6	nonsynonymous SNV
chr9	135546107	C	T	22	26	48	GTF3C4	nonsynonymous SNV
chr9	136308659	C	A	26	35	61	ADAMTS13	stopgain
chr10	45487384	T	A	39	33	72	RASSF4	nonsynonymous SNV

chr10	63976944	T	TA	43	34	77	RTKN2	frameshift insertion
chr10	76780901	G	A	63	55	118	KAT6B	nonsynonymous SNV
chr10	101180556	C	A	89	76	165	GOT1	nonsynonymous SNV
chr10	105139754	A	G	48	35	83	TAF5	nonsynonymous SNV
chr11	1651120	G	A	81	47	128	KRTAP5-5	nonsynonymous SNV
chr11	5664767	G	A	48	77	125	TRIM34,TRIM6-TRIM34	nonsynonymous SNV
chr11	34154640	C	T	144	136	280	NAT10	nonsynonymous SNV
chr11	46771866	C	T	66	48	114	CKAP5	nonsynonymous SNV
chr11	57068427	G	A	106	105	211	TNKS1BP1	nonsynonymous SNV
chr11	64976805	C	T	114	99	213	CAPN1	nonsynonymous SNV
chr11	66834227	G	A	95	125	220	RHOD	nonsynonymous SNV
chr11	78369177	C	T	266	266	532	TENM4	nonsynonymous SNV
chr11	99690483	C	CAACTGAGGAACCAGGCATTATTTTGTCGATAGATCCAAAATTGACAAAGGTAGACAACATCTAGAAAATATTA	108	26	134	CNTN5	stopgain
chr11	133790496	G	A	116	163	279	IGSF9B	nonsynonymous SNV
chr12	27654916	T	A	26	34	60	SMCO2	stopgain

chr12	55968659	T	C	47	28	75	OR2AP1	nonsynonymous SNV
chr12	122261544	G	A	308	289	597	SETD1B	nonsynonymous SNV
chr12	128899814	C	T	176	239	415	TMEM132C	nonsynonymous SNV
chr13	20412874	G	A	41	43	84	ZMYM5	nonsynonymous SNV
chr13	25670801	T	TG	72	14	86	PABPC3	frameshift insertion
chr13	103410908	T	G	22	30	52	CCDC168	nonsynonymous SNV
chr14	57755591	T	G	32	31	63	AP5M1	nonsynonymous SNV
chr14	73721320	C	T	190	181	371	PAPLN	nonsynonymous SNV
chr14	92537354	C	CTGCTGCTGCTGCTGCTGCTGCTGCTGCTGCTGCTGCTGCTG	40	30	70	ATXN3	frameshift insertion
chr14	96778440	A	T	21	27	48	ATG2B	nonsynonymous SNV
chr14	104641674	G	T	264	285	549	KIF26A	nonsynonymous SNV
chr15	23891563	C	G	19	22	41	MAGEL2	nonsynonymous SNV
chr15	42375440	G	A	94	84	178	PLA2G4D	nonsynonymous SNV
chr15	50519297	A	G	59	56	115	SLC27A2	nonsynonymous SNV
chr15	63632575	C	G	129	121	250	CA12	nonsynonymous SNV
chr15	72106440	A	G	189	172	361	NR2E3	nonsynonymous SNV
chr15	90334274	G	A	177	180	357	ANPEP	nonsynonymous SNV
chr15	94882594	T	G	33	26	59	MCTP2	nonsynonymous SNV
chr16	747321	C	T	76	69	145	FBXL16	nonsynonymous SNV
chr16	19800212	CT	C	60	75	135	IQCK	frameshift deletion

chr16	19800214	CTA	C	60	75	135	IQCK	frameshift deletion
chr16	28944321	G	A	117	94	211	CD19	nonsynonymous SNV
chr16	31153925	C	CA	111	99	210	PRSS36	frameshift insertion
chr16	70524268	T	G	92	62	154	COG4	nonsynonymous SNV
chr17	11701106	C	T	68	56	124	DNAH9	nonsynonymous SNV
chr17	17898353	A	G	58	91	149	DRC3	nonsynonymous SNV
chr17	48745198	C	T	132	90	222	ABCC3	nonsynonymous SNV
chr17	56396645	C	T	48	43	91	BZRAP1	nonsynonymous SNV
chr17	61566047	G	A	144	112	256	ACE	nonsynonymous SNV
chr17	61621448	TG	T	63	60	123	KCNH6	frameshift deletion
chr17	73096632	G	T	197	187	384	SLC16A5	nonsynonymous SNV
chr17	78360175	C	T	122	84	206	RNF213	nonsynonymous SNV
chr18	5478433	G	A	59	70	129	EPB41L3	nonsynonymous SNV
chr18	6966151	C	T	16	29	45	LAMA1	nonsynonymous SNV
chr18	56585881	T	A	234	218	452	ZNF532	nonsynonymous SNV
chr18	76753658	C	T	147	161	308	SALL3	nonsynonymous SNV
chr19	15853084	AAGGAACAAGG	A	26	16	42	OR10H3	frameshift deletion
chr19	17397491	GTGTGTGTGTT	G	93	25	118	ANKLE1	frameshift deletion
chr19	38673323	G	C	76	51	127	SIPA1L3	nonsynonymous SNV
chr19	39019691	A	G	24	33	57	RYR1	nonsynonymous SNV
chr19	50062160	C	G	82	67	149	NOSIP	nonsynonymous SNV
chr19	55610381	C	T	88	108	196	PPP1R12C	nonsynonymous SNV
chr19	59073888	G	C	164	139	303	MZF1	nonsynonymous SNV

chr20	861929	G	T	176	160	336	ANGPT4	nonsynonymous SNV
chr20	5904099	G	C	58	44	102	CHGB	nonsynonymous SNV
chr20	47864591	A	G	46	43	89	ZNFX1	nonsynonymous SNV
chr21	34837684	C	A	24	33	57	TMEM50B	nonsynonymous SNV
chr21	47704374	T	G	90	93	183	MCM3AP	nonsynonymous SNV
chr22	20136295	C	T	68	45	113	CCDC188	nonsynonymous SNV
chr22	31524552	G	A	165	133	298	INPP5J	nonsynonymous SNV
chr22	36876668	G	A	134	125	259	TXN2	stopgain
chr22	39909585	T	C	112	119	231	MIEF1	nonsynonymous SNV
chr22	43568527	A	G	220	171	391	TTLL12	nonsynonymous SNV
chrX	35988941	C	A	24	18	42	CFAP47	nonsynonymous SNV
chrX	114424822	G	A	245	243	488	RBMXL3	nonsynonymous SNV
chrX	153590884	C	T	165	190	355	FLNA	nonsynonymous SNV
**Patient 73**								
**Chromosome**	**Position**	**REF**	**ALT**	**REF-depth**	**ALT-depth**	**Total-depth**	**Gene**	**ExonicFunc.refGene**
chr1	45506178	C	T	47	37	84	ZSWIM5	nonsynonymous SNV
chr1	53662713	A	G	118	119	237	CPT2	nonsynonymous SNV
chr1	66102145	C	A	72	85	157	LEPR	nonsynonymous SNV
chr1	109777919	C	A	92	69	161	SARS	nonsynonymous SNV
chr1	151341587	C	T	92	116	208	SELENBP1	nonsynonymous SNV
chr1	151773718	T	C	220	230	450	LINGO4	nonsynonymous SNV
chr1	182025707	T	C	247	216	463	ZNF648	nonsynonymous SNV
chr1	201195129	G	A	249	199	448	IGFN1	nonsynonymous SNV
chr1	222750852	T	C	49	48	97	TAF1A	nonsynonymous SNV

chr1	231298986	G	T	84	102	186	TRIM67	nonsynonymous SNV
chr2	38800446	G	C	58	45	103	HNRNPLL	nonsynonymous SNV
chr2	80540729	T	G	62	59	121	CTNNA2	nonsynonymous SNV
chr2	88474312	C	G	98	100	198	THNSL2	stopgain
chr2	102486868	C	G	43	30	73	MAP4K4	nonsynonymous SNV
chr2	135890552	T	C	32	14	46	RAB3GAP1	nonsynonymous SNV
chr2	152235992	G	GT	22	29	51	TNFAIP6	frameshift insertion
chr2	219676968	T	G	141	108	249	CYP27A1	nonsynonymous SNV
chr2	224765921	G	A	85	72	157	WDFY1	nonsynonymous SNV
chr2	228162406	G	T	17	12	29	COL4A3	nonsynonymous SNV
chr2	236839502	A	G	136	137	273	AGAP1	nonsynonymous SNV
chr3	46307677	C	T	64	56	120	CCR3	nonsynonymous SNV
chr3	120973753	A	G	34	32	66	STXBP5L	nonsynonymous SNV
chr3	126261062	G	T	173	147	320	CHST13	nonsynonymous SNV
chr4	998093	A	C	148	124	272	IDUA	nonsynonymous SNV
chr4	2444929	C	T	295	257	552	CFAP99	nonsynonymous SNV
chr4	8288409	C	T	156	129	285	HTRA3	stopgain
chr4	71063798	C	T	44	58	102	ODAM	nonsynonymous SNV
chr4	71705447	T	G	64	61	125	GRSF1	nonsynonymous SNV
chr4	144141592	G	C	72	43	115	USP38	nonsynonymous SNV
chr4	169327824	G	C	21	15	36	DDX60L	nonsynonymous SNV
chr4	187130250	G	T	156	123	279	CYP4V2	nonsynonymous SNV
chr5	5461864	G	C	52	34	86	ICE1	nonsynonymous SNV
chr5	121515294	G	A	29	22	51	LOC100505	nonsynonymous SNV

							841	
chr6	10621625	C	T	77	52	129	GCNT2	nonsynonymous SNV
chr6	11538523	G	C	103	91	194	TMEM170B	nonsynonymous SNV
chr6	20739793	G	A	76	47	123	CDKAL1	nonsynonymous SNV
chr6	26108264	G	A	91	86	177	HIST1H1T	nonsynonymous SNV
chr6	51892960	G	A	56	75	131	PKHD1	nonsynonymous SNV
chr6	55922520	C	T	112	103	215	COL21A1	nonsynonymous SNV
chr6	56365958	G	A	45	46	91	DST	nonsynonymous SNV
chr6	66204918	C	A	56	46	102	EYS	nonsynonymous SNV
chr6	83841983	A	C	40	22	62	DOPEY1	nonsynonymous SNV
chr6	106960689	C	G	64	87	151	AIM1	nonsynonymous SNV
chr6	109477028	T	G	31	31	62	CEP57L1	nonsynonymous SNV
chr6	135360884	T	C	42	52	94	HBS1L	nonsynonymous SNV
chr6	144744727	G	A	91	90	181	UTRN	nonsynonymous SNV
chr7	286468	G	GGACAGGTG AGCCCTTCCT TCCTCCCTCC ATCCGC	55	47	102	FAM20C	stopgain
chr7	6189425	G	T	41	37	78	USP42	nonsynonymous SNV
chr7	26686061	G	T	61	27	88	C7orf71	nonsynonymous SNV
chr7	86574346	G	A	55	40	95	KIAA1324L	nonsynonymous SNV
chr7	97937038	C	T	49	62	111	BAIAP2L1	nonsynonymous SNV
chr7	100421405	C	T	175	170	345	EPHB4	nonsynonymous SNV
chr7	102953519	T	C	45	18	63	DNAJC2	nonsynonymous SNV

chr8	1851528	G	A	14	31	45	ARHGEF10	nonsynonymous SNV
chr8	12947954	C	G	50	44	94	DLC1	nonsynonymous SNV
chr8	37702609	C	T	108	108	216	BRF2	nonsynonymous SNV
chr8	49643948	T	C	94	75	169	EFCAB1	nonsynonymous SNV
chr8	59409637	A	C	61	84	145	CYP7A1	nonsynonymous SNV
chr9	72003143	T	C	80	66	146	FAM189A2	nonsynonymous SNV
chr9	72897491	A	G	11	9	20	SMC5	nonsynonymous SNV
chr9	73426095	C	A	51	29	80	TRPM3	nonsynonymous SNV
chr9	95076749	G	C	74	76	150	NOL8	nonsynonymous SNV
chr9	96259819	C	T	56	39	95	FAM120A	nonsynonymous SNV
chr9	96864014	C	G	36	45	81	PTPDC1	nonsynonymous SNV
chr9	109691319	G	A	231	202	433	ZNF462	nonsynonymous SNV
chr9	123177341	C	T	48	25	73	CDK5RAP2	nonsynonymous SNV
chr9	131589450	C	T	80	102	182	C9orf114	nonsynonymous SNV
chr9	139369087	A	G	88	90	178	SEC16A	nonsynonymous SNV
chr9	139656276	G	A	31	19	50	LCN15	nonsynonymous SNV
chr9	140065569	G	A	152	129	281	TMEM210	nonsynonymous SNV
chr10	16882957	G	T	23	17	40	CUBN	nonsynonymous SNV
chr10	73058904	C	T	172	111	283	UNC5B	nonsynonymous SNV
chr10	73572052	C	T	137	164	301	CDH23	nonsynonymous SNV
chr11	1957456	C	T	163	149	312	TNNT3	nonsynonymous SNV
chr11	8132446	A	T	70	74	144	RIC3	stopgain
chr11	12023911	T	C	138	150	288	DKK3	nonsynonymous SNV
chr11	17569088	G	T	35	34	69	OTOG	nonsynonymous SNV

chr11	61110874	T	C	178	127	305	TKFC	nonsynonymous SNV
chr11	67191687	G	A	286	196	482	CARNS1	nonsynonymous SNV
chr11	96125283	T	A	40	37	77	JRKL	nonsynonymous SNV
chr11	108235872	CAGAGGCCGGA	C	61	26	87	ATM	frameshift deletion
chr11	108235887	G	GT	60	27	87	ATM	frameshift insertion
chr11	117099032	A	G	122	126	248	PCSK7	nonsynonymous SNV
chr12	52376515	C	T	79	71	150	ACVR1B	nonsynonymous SNV
chr12	77423985	C	A	72	51	123	E2F7	nonsynonymous SNV
chr12	81239653	C	A	46	43	89	LIN7A	nonsynonymous SNV
chr12	101767208	C	T	115	77	192	UTP20	nonsynonymous SNV
chr12	109872843	A	G	54	49	103	MYO1H	nonsynonymous SNV
chr12	125441612	C	T	119	195	314	DHX37	nonsynonymous SNV
chr13	114778701	C	G	172	146	318	RASA3	nonsynonymous SNV
chr14	21553915	G	A	62	72	134	ARHGEF40	nonsynonymous SNV
chr14	73407009	C	T	146	128	274	DCAF4	nonsynonymous SNV
chr14	104570692	G	C	73	68	141	ASPG	nonsynonymous SNV
chr15	22855219	A	G	126	132	258	TUBGCP5	nonsynonymous SNV
chr15	32685308	G	A	38	22	60	GOLGA8K	nonsynonymous SNV
chr15	35274008	T	C	15	20	35	ZNF770	nonsynonymous SNV
chr15	43815794	G	A	96	72	168	MAP1A	nonsynonymous SNV
chr15	45401724	C	T	108	63	171	DUOX2	nonsynonymous SNV
chr16	30206233	C	G	25	6	31	SLX1A,SLX1B	nonsynonymous SNV
chr16	31091378	C	T	303	243	546	ZNF646	nonsynonymous SNV

chr16	31475767	G	A	285	302	587	ARMC5	nonsynonymous SNV
chr16	67034616	C	CAGCGAGGA CTGTCTGTGA	62	46	108	CES4A	stopgain
chr16	67324838	C	T	112	113	225	KCTD19	nonsynonymous SNV
chr16	78198137	G	T	89	93	182	WWOX	nonsynonymous SNV
chr17	5358558	C	T	55	56	111	DHX33	nonsynonymous SNV
chr17	9683193	C	T	71	65	136	DHRS7C	nonsynonymous SNV
chr17	27493313	G	A	208	162	370	MYO18A	nonsynonymous SNV
chr17	40312080	G	T	39	19	58	KCNH4	nonsynonymous SNV
chr17	40330149	C	T	107	71	178	KCNH4	nonsynonymous SNV
chr17	74536225	T	C	175	178	353	PRCD	nonsynonymous SNV
chr17	79869164	A	G	58	45	103	PCYT2	nonsynonymous SNV
chr17	79899320	C	T	153	147	300	MYADML2	nonsynonymous SNV
chr17	79980710	A	C	68	75	143	STRA13	nonsynonymous SNV
chr18	29052367	G	A	42	51	93	DSG3	nonsynonymous SNV
chr18	29782861	A	G	12	20	32	MEP1B	nonsynonymous SNV
chr18	44068983	C	T	130	117	247	LOXHD1	nonsynonymous SNV
chr18	47432897	A	T	183	191	374	MYO5B	nonsynonymous SNV
chr19	5135465	C	T	224	259	483	KDM4B	nonsynonymous SNV
chr19	7964465	T	C	280	218	498	LRRC8E	nonsynonymous SNV
chr19	12936536	C	T	28	38	66	RTBDN	nonsynonymous SNV
chr19	13201118	G	C	264	266	530	NFIX	nonsynonymous SNV
chr19	21132044	T	C	65	64	129	ZNF85	nonsynonymous SNV
chr19	38189887	C	G	36	31	67	ZNF607	nonsynonymous SNV

chr19	42354435	C	T	127	106	233	DMRTC2	nonsynonymous SNV
chr19	47551757	G	C	27	21	48	TMEM160	nonsynonymous SNV
chr19	50404959	A	G	169	109	278	IL4I1	nonsynonymous SNV
chr19	58907743	C	A	117	156	273	RNF225	stopgain
chr20	32255349	A	T	72	62	134	ACTL10	nonsynonymous SNV
chr20	35433227	G	A	134	169	303	SOGA1	nonsynonymous SNV
chr20	41514536	C	A	86	54	140	PTPRT	nonsynonymous SNV
chr20	49626503	C	A	264	225	489	KCNG1	nonsynonymous SNV
chr21	45564737	C	T	137	171	308	C21orf33	nonsynonymous SNV
chr22	20103296	C	T	26	32	58	TRMT2A	nonsynonymous SNV
chr22	22869648	C	T	89	91	180	ZNF280A	nonsynonymous SNV
**Patient 114**								
**Chromosome**	**Position**	**REF**	**ALT**	**REF-depth**	**ALT-depth**	**Total-depth**	**Gene**	**ExonicFunc.refGene**
chr1	15988179	C	G	64	52	116	RSC1A1	nonsynonymous SNV
chr1	21031201	G	A	322	259	581	KIF17	nonsynonymous SNV
chr1	26073271	C	A	117	94	211	MAN1C1	nonsynonymous SNV
chr1	43778060	C	T	207	216	423	TIE1	nonsynonymous SNV
chr1	45293263	T	A	131	116	247	PTCH2	nonsynonymous SNV
chr1	147092212	C	T	62	81	143	BCL9	nonsynonymous SNV
chr1	154305134	T	C	38	22	60	ATP8B2	nonsynonymous SNV
chr1	156641983	G	A	31	58	89	NES	nonsynonymous SNV
chr1	163138150	T	C	29	32	61	RGS5	nonsynonymous SNV
chr1	172502516	G	A	78	74	152	SUCO	nonsynonymous SNV
chr1	173915954	G	A	11	17	28	RC3H1	nonsynonymous SNV

chr1	215259899	G	A	113	96	209	KCNK2	nonsynonymous SNV
chr1	226923379	G	A	114	106	220	ITPKB	nonsynonymous SNV
chr1	228463715	C	T	205	219	424	OBSCN	nonsynonymous SNV
chr1	230928178	G	A	62	83	145	CAPN9	nonsynonymous SNV
chr2	44202246	A	T	44	38	82	LRPPRC	nonsynonymous SNV
chr2	55074693	T	A	77	119	196	EML6	nonsynonymous SNV
chr2	99012982	C	T	187	139	326	CNGA3	nonsynonymous SNV
chr2	99438401	G	T	57	52	109	KIAA1211L	nonsynonymous SNV
chr2	179396824	G	A	78	78	156	TTN	nonsynonymous SNV
chr2	179664286	T	C	113	111	224	TTN	nonsynonymous SNV
chr2	182339687	G	A	39	13	52	ITGA4	nonsynonymous SNV
chr2	201721696	T	C	43	36	79	CLK1	nonsynonymous SNV
chr2	220174020	C	T	27	35	62	PTPRN	nonsynonymous SNV
chr2	232389951	G	A	143	132	275	NMUR1	nonsynonymous SNV
chr2	239010703	G	A	83	92	175	ESPNL	nonsynonymous SNV
chr3	33183901	A	C	80	74	154	CRTAP	nonsynonymous SNV
chr3	52398873	C	T	211	162	373	DNAH1	nonsynonymous SNV
chr3	63898516	G	A	129	105	234	ATXN7	nonsynonymous SNV
chr3	71830622	C	T	32	22	54	PROK2	nonsynonymous SNV
chr3	72799943	G	C	10	15	25	SHQ1	nonsynonymous SNV
chr3	120050139	C	T	60	66	126	LRRC58	nonsynonymous SNV
chr3	122645437	G	A	167	139	306	SEMA5B	nonsynonymous SNV
chr3	124351375	G	A	107	109	216	KALRN	nonsynonymous SNV
chr3	129127601	T	A	181	181	362	EFCAB12	nonsynonymous SNV

chr5	96245365	C	G	69	95	164	ERAP2	nonsynonymous SNV
chr5	126862349	C	T	58	24	82	PRRC1	nonsynonymous SNV
chr5	134782548	C	T	152	149	301	DCANP1	nonsynonymous SNV
chr5	140249740	T	C	138	119	257	PCDHA11	nonsynonymous SNV
chr5	145465086	G	A	81	45	126	PLAC8L1	nonsynonymous SNV
chr5	156522440	C	G	41	41	82	HAVCR2	nonsynonymous SNV
chr5	159680777	C	T	91	56	147	CCNJL	nonsynonymous SNV
chr5	161116765	A	C	49	42	91	GABRA6	nonsynonymous SNV
chr6	11190918	G	A	153	111	264	NEDD9	nonsynonymous SNV
chr6	76412570	G	A	51	37	88	SENP6	nonsynonymous SNV
chr6	84799150	G	T	77	73	150	MRAP2	nonsynonymous SNV
chr6	138645202	G	A	123	115	238	ARFGEF3	nonsynonymous SNV
chr6	138725652	C	T	10	16	26	HEBP2	nonsynonymous SNV
chr7	330753	G	T	241	258	499	WI2-2373I1.2	nonsynonymous SNV
chr7	75615259	C	T	72	54	126	POR	nonsynonymous SNV
chr7	101988786	G	A	33	6	39	SPDYE6	nonsynonymous SNV
chr7	140301263	G	C	92	79	171	DENND2A	nonsynonymous SNV
chr8	2007334	C	G	181	151	332	MYOM2	nonsynonymous SNV
chr8	30038093	A	G	29	30	59	DCTN6	nonsynonymous SNV
chr8	123964153	G	A	123	92	215	ZHX2	nonsynonymous SNV
chr8	145623597	ATGCCCAGGGCCCAACCACCTCCCCCCCACCGTCCCCA	A	119	97	216	CPSF1	frameshift deletion

		CCCACCTACCTCC TTCCAGCAGGCG GATGCCCAGGGC CCAACCACCCCCC CACCTACCTCCTT CCAGCAG						
chr9	396802	G	C	50	52	102	DOCK8	nonsynonymous SNV
chr9	33264660	C	A	7	6	13	BAG1	nonsynonymous SNV
chr9	34517463	A	G	98	85	183	DNAI1	nonsynonymous SNV
chr9	40705251	C	T	12	11	23	SPATA31A 3	nonsynonymous SNV
chr9	99250384	A	C	216	220	436	HABP4	nonsynonymous SNV
chr9	125288974	A	C	102	111	213	OR1N1	nonsynonymous SNV
chr9	130889962	C	T	12	13	25	PTGES2	stopgain
chr10	5810340	C	T	66	70	136	GDI2	nonsynonymous SNV
chr10	14816527	C	A	164	160	324	FAM107B	nonsynonymous SNV
chr10	21435331	A	G	61	39	100	C10orf113	nonsynonymous SNV
chr10	55943338	C	T	49	54	103	PCDH15	nonsynonymous SNV
chr10	61833083	T	C	39	44	83	ANK3	nonsynonymous SNV
chr10	73472553	G	A	110	113	223	CDH23	nonsynonymous SNV
chr10	79581231	G	T	81	59	140	DLG5	nonsynonymous SNV
chr10	86007377	C	T	190	145	335	RGR	nonsynonymous SNV
chr10	120795730	G	A	111	98	209	EIF3A	nonsynonymous SNV
chr11	554265	G	C	123	108	231	LRRC56	nonsynonymous SNV

chr11	1249954	C	T	22	16	38	MUC5B	nonsynonymous SNV
chr11	62296580	T	C	69	84	153	AHNAK	nonsynonymous SNV
chr11	62298881	A	G	42	82	124	AHNAK	nonsynonymous SNV
chr11	66281997	A	G	264	230	494	BBS1	nonsynonymous SNV
chr11	67176957	C	T	83	71	154	TBC1D10C	nonsynonymous SNV
chr11	73007750	G	A	154	142	296	P2RY6	nonsynonymous SNV
chr11	73073523	G	T	69	70	139	ARHGEF17	nonsynonymous SNV
chr11	74345721	C	G	49	48	97	POLD3	nonsynonymous SNV
chr11	119053051	T	C	118	102	220	NLRX1	nonsynonymous SNV
chr12	7653947	A	G	145	124	269	CD163	nonsynonymous SNV
chr12	11420391	TGGACGAGGTGGGGGACCTTGGGACTGGTTTCCTCCTTGTGGGGGTGGTCCTTCTGGCTTTCCTGGACGAGGTGGGGGACCTTGAGGTTTGTTGCCTCCTTGTGGGGGTGGTCCTTCTGGCTTTCCC	T	36	153	189	PRB3	frameshift deletion
chr12	45741891	CA	C	80	80	160	ANO6	frameshift deletion
chr12	49459186	G	C	108	88	196	RHEBL1	nonsynonymous SNV
chr12	50393435	TCTCAATAGTGGACACAG	T	51	13	64	RACGAP1	frameshift deletion

chr12	50393461	G	T	49	12	61	RACGAP1	nonsynonymous SNV
chr12	54963379	G	C	85	72	157	PDE1B	nonsynonymous SNV
chr12	56530556	T	C	43	31	74	ESYT1	nonsynonymous SNV
chr12	59271518	C	T	101	112	213	LRIG3	nonsynonymous SNV
chr12	109181985	G	A	237	251	488	SSH1	nonsynonymous SNV
chr12	111956157	G	A	55	36	91	ATXN2	nonsynonymous SNV
chr12	131593398	C	T	151	123	274	ADGRD1	nonsynonymous SNV
chr13	22178145	C	A	81	82	163	MICU2	nonsynonymous SNV
chr13	39446997	G	A	60	62	122	FREM2	nonsynonymous SNV
chr13	41834900	G	T	40	53	93	MTRF1	stopgain
chr15	41796358	A	G	97	79	176	LTK	nonsynonymous SNV
chr15	75651973	C	A	134	133	267	MAN2C1	nonsynonymous SNV
chr15	78894437	C	T	157	171	328	CHRNA3	nonsynonymous SNV
chr15	86312658	CT	C	175	157	332	KLHL25	frameshift deletion
chr15	89848426	C	T	64	83	147	FANCI	nonsynonymous SNV
chr16	28855117	C	T	50	28	78	TUFM	nonsynonymous SNV
chr16	30793073	A	C	74	93	167	ZNF629	nonsynonymous SNV
chr16	84063142	G	T	51	54	105	SLC38A8	nonsynonymous SNV
chr17	1387467	C	T	205	201	406	MYO1C	nonsynonymous SNV
chr17	3518631	C	A	127	104	231	SHPK	nonsynonymous SNV
chr17	26818587	C	T	183	187	370	SLC13A2	nonsynonymous SNV
chr17	36704803	G	A	81	66	147	SRCIN1	nonsynonymous SNV
chr17	57093077	T	C	73	75	148	TRIM37	nonsynonymous SNV
chr17	79165039	C	T	261	244	505	CEP131	nonsynonymous SNV

chr18	77246317	G	A	123	83	206	NFATC1	nonsynonymous SNV
chr19	1122948	G	A	80	79	159	SBNO2	nonsynonymous SNV
chr19	3002383	C	T	78	55	133	TLE2	nonsynonymous SNV
chr19	10201932	C	T	128	80	208	C19orf66	nonsynonymous SNV
chr19	12429928	CTG	C	113	101	214	ZNF563	frameshift deletion
chr19	18184332	C	T	64	52	116	IL12RB1	nonsynonymous SNV
chr19	36884764	G	A	29	51	80	ZFP82	nonsynonymous SNV
chr19	44096434	C	A	93	144	237	IRGQ	nonsynonymous SNV
chr19	52568449	G	A	47	34	81	ZNF841	stopgain
chr19	57089347	C	T	54	36	90	ZNF470	nonsynonymous SNV
chr20	278240	G	A	46	32	78	ZCCHC3	nonsynonymous SNV
chr20	825896	C	T	108	72	180	FAM110A	nonsynonymous SNV
chr20	47887288	C	T	62	59	121	ZNFX1	nonsynonymous SNV
chr20	48600655	A	G	203	193	396	SNAI1	nonsynonymous SNV
chr20	62373525	G	A	114	85	199	SLC2A4RG	nonsynonymous SNV
chr21	46000352	G	C	77	94	171	KRTAP10-5	nonsynonymous SNV
chr22	18022041	C	T	149	156	305	CECR2	nonsynonymous SNV
chr22	29446636	C	G	146	160	306	ZNRF3	nonsynonymous SNV
chr22	37414320	C	A	120	96	216	TST	nonsynonymous SNV
chr22	41257857	C	T	42	54	96	DNAJB7	nonsynonymous SNV
chr22	42992367	C	T	73	76	149	POLDIP3	nonsynonymous SNV
chr22	50965115	A	G	114	65	179	TYMP	nonsynonymous SNV
chrX	19379697	G	C	40	38	78	MAP3K15	nonsynonymous SNV
chrX	32563365	C	A	58	50	108	DMD	nonsynonymous SNV

chrX	48419133	C	T	196	211	407	TBC1D25	nonsynonymous SNV
chrX	104464853	G	A	91	74	165	TEX13A	nonsynonymous SNV
chrX	153039131	C	A	48	18	66	PLXNB3	nonsynonymous SNV
chrX	153176191	G	T	93	86	179	ARHGAP4	nonsynonymous SNV
