# Supplementary material for: TAMM41 is required for heart valve differentiation via regulation of PINK-PARK2 dependent mitophagy
Source: Cell Death Differ. 2019 Mar 1;26(11):2430–46. doi: 10.1038/s41418-019-0311-z (PMC6888875; doi:10.1038/s41418-019-0311-z)
Supplement: Supplementary file 4 — Supplemental table 3 [file 41418_2019_311_MOESM4_ESM.pdf]

Table S3. Detailed characterizations of the phenotypes of the CHD patients collected.

No.	Gender	Date of Born	Diagnosis
1	Female	2012.9	CAVC-A, LSVC, PH
2	Female	2012.10	CAVC-A, ASD, PH
3	Male	2012.10	CAVC-A, ASD, PH
4	Male	2012.12	CAVC-A, ASD, PH
5	Female	2010.3	CAVC-A, PH
6	Female	2010.11	CAVC-C, PH
7	Male	2010.11	CAVC-A, ASD, PH
8	Male	2012.7	CAVC-A, PH
9	Female	2010.11	CAVC-A, PH
10	Male	2005.3	CAVC-A, PH
11	Male	2012.5	CAVC-A, PH
12	Female	2012.9	CAVC-A, PH
13	Male	2010.8	CAVC-A, PH
14	Female	2006.5	CAVC-C, ASD, PDA, PH
15	Female	2006.9	CAVC-A, PH
16	Female	2010.5	CAVC-A, ASD, PDA, PH
17	Female	2010.10	CAVC-A, H
18	Female	2010.3	CAVC-A, PH
19	Male	2009.1	CAVC-C, ASD, LSVC,PH
20	Male	2010.10	CAVC-A, ASD,PH
21	Female	2012.7	CAVC-B, ASD, PDA,PH
22	Male	2011.9	CAVC-A, PH
23	Female	2009.11	CAVC-A, ASD, PH
24	Male	2011.5	CAVC-A, PH
25	Female	2012.10	CAVC-A, PH
26	Male	2010.8	CAVC-A, ASD, PH
27	Male	2012.2	CAVC-A, ASD, PH
28	Male	2011.4	CAVC-A, ASD, PH
29	Male	2010.5	CAVC-A, PH
30	Female	2010.3	CAVC-A, PH
31	Male	2009.11	CAVC-A, PH
32	Male	2012.7	CAVC-A, PH
33	Female	2010.8	TAVC
34	Female	2002.11	CAVC-A, PH
35	Male	2012.2	CAVC-B, PH
36	Female	2009.11	CAVC-A, ASD, PDA, PH
37	Male	2010.7	CAVC-A, PH
38	Male	2010.8	CAVC-A, PH
39	Male	2007.3	SV, CAVC, SA, PS, TAPVC

40	Male	2011.10	TAVC, ASD, PH
41	Female	2009.11	CAVC-A, PH
42	Male	2010.1	CAVC-A, PH
43	Male	2011.11	CAVC-A, PH
44	Male	2011.10	TAVC
45	Male	2010.9	CAVC-A, PH
46	Female	2012.2	CAVC-A, PH
47	Male	2012.9	CAVC-A, PH
48	Male	2012.3	CAVC-A, PH
49	Female	2010.6	CAVC-A, PH
50	Female	2007.10	CAVC-A, PH
51	Female	2006.10	DORV/ CAVC-C, TAPVC, PDA, PS
52	Female	2007.7	DORV/CAVC, PS
53	Female	2009.11	TOF/CAVC-A, LSVC
54	Male	2010.5	DORV/CAVC-C, TAPVC, PDA, PS
55	Male	2006.7	SV, CAVC, SA, PS, PDA
56	Male	2003.9	SV, CAVC, SA, PS, LSVC
57	Female	2009.4	DORV, CAVC, SA, PS, PDA, LSVC
58	Male	2007.3	SV, PA, CAVC, PDA
59	Female	2008.5	RH，SV(R), MGA, PS, CAVC, Double superior vena cava
60	Male	2010.7	DORV, VSD，CAVC,PH
61	Male	2010.4	SV（R），SA，MGA，PS，PAPVC
62	Male	2008.10	SV（R），ASD，PS
63	Male	2010.6	DORV, CAVC, PS, DSVC
64	Male	2009.2	DORV（S、D、D），CAVC ，SA，PS
65	Female	2001.9	D-TGA，CAVC,ASD,PS
66	Female	2008.5	RH，DORV，CAVC,，SA,PS,DSVC
67	Female	2010.12	SV（L），MGA，PS，CAVC,TAPVC
68	Female	2009.2	TOF，CAVC，PDA
69	Female	2006.7	Middle heart, DORV, CAVC, PS, SA, PDA, TAPVC
70	Female	2011.5	SA, SV, TAPVC, PH
71	Female	2011.2	DORV (A,D,D), CAVC, SA, PS, DSVC
72	Male	2010.12	L-TGA (A,L,L), CAVC, PS, CAVC, ASD
73	Male	2008.6	SV(R), MGA, PS, PDA, CAVC,
74	Male	1998.9	PA, CAVC, ASD
75	Male	2011.2	TOF, CAVC
76	Female	2011.1	DORV, CAVC, SA, PS, PDA, DSVC
77	Female	2010.11	TOF, CAVC
78	Male	2010.2	LSVC, DORV, VSD, PS, PDA, SA
79	Male	2008.11	DORV(A,D,D), VSD，CAVC, PS, SA
80	Male	2011.3	SV, MGA, PS, TAPVC
81	Male	2001.4	SV, CAVC, PS

82	Male	2005.10	CAVC-A, PDA, PH
83	Male	2008.8	DORV (S,D,D), VSD, ASD, PS
84	Female	2007.9	SV(R), MGA, SA, PS, PDA
85	Male	2011.11	SV(R), CAVC, MGA, PS, SA, TAPVC
86	Female	2011.9	DORV(A,D,A), VSD, ASD, PS, TAPVC
87	Female	2003.2	DORV, CAVC, VSD, ASD, PS
88	Male	2003.9	DORV(A,D,D)， VSD, CAVC, PS, PDA
89	Female	1997.11	RH, DORV, PS, CAVC, ASD
90	Male	2007.3	SA, SV, PS, CAVC
91	Female	2012.5	PAVC, ASD, PDA, PH
92	Male	2012.4	DORV, CAVC, PS
93	Male	2009.5	TOF, CAVC, TAPVC
94	Male	2006.5	RH, DORV, CAVC, SA, PS
95	Male	2012.4	RH， SV(R), MGA, ASD, CAVC, PH
96	Male	2012.10	DORV (A,D,D), VSD, PS, TAPVC, CAVC
97	Male	2012.8	SV(R), PA,PDA, CAVC, PAPVC
98	Female	2012.5	CAVC-A,PH， LSVC
99	Female	1999.9	CAVC-A, PH
100	Male	2011.10	DORV, CAVC, PS, PDA
101	Male	2012.11	CAVC-A, PH
102	Female	2010.4	CAVC-A, PH
103	Female	2010.5	PA/VSD， PDA， CAVC，
104	Female	2012.11	CAVC-A, ASD, PDA, PH
105	Male	2012.8	RH， DORV(A,L,L)， CAVC,PDA,CAV, PH
106	Male	2010.5	CAVC-A, PH
107	Male	2009.10	RH, DORV, SA, CAVC, PS
108	Female	2012.9	CAVC-A, PH
109	Female	2012.10	CAVC-A, PH
110	Female	2014.6	CAVC-A, PH, CoA,PDA
111	Female	2012.12	CAVC-A, PH
112	Female	2012.11	CAVC-A, PDA, PH
113	Male	2012.9	CAVC-A, PH
114	Female	2012.12	CAVC-A, PH
115	Male	2012.12	D-TGA,PA, PDA, CAVC, ASD
116	Male	2005.6	CAVC-A, PH
117	Male	2006.10	CAVC-C, VSD, PDA, SA, PH
118	Male	2010.6	RH， DORV(A,L,A), CAVC, SA, PS

ASD, Atrial Septal Defect; CAVC, Complete Atrio-Ventricular Septal Defect; CoA, Aortic Coarctation; DORV, Double Outlet of Right Ventricle; D-TGA, Complete Transposition of Great Arteries; IVC, Inferior Vena Cava; L-TGA, Corrective

Transposition of Great Arteries; LSVC, Left Superior Vena Cava; MGA, Large Artery Dislocation; PA, Pulmonary Atresia; PAVC, Partial Atrio-Ventricular Septal Defect; PAPVC, Partial Anomalous Pulmonary Venous Connection; PH, Pulmonary Hypertension; PDA, Patent Ductus Arteriosus; PS, Pulmonary Artery Stenosis; SV, Single Ventricle; TAPVC, Total Anomalous Pulmonary Venous Connection; TOF, Tetralogy of Fallot; RH, Right Heart.
