# Supplementary material for: TAMM41 is required for heart valve differentiation via regulation of PINK-PARK2 dependent mitophagy
Source: Cell Death Differ. 2019 Mar 1;26(11):2430–46. doi: 10.1038/s41418-019-0311-z (PMC6888875; doi:10.1038/s41418-019-0311-z)
Supplement: Supplementary file 5 — Supplemental table 4 [file 41418_2019_311_MOESM5_ESM.pdf]

Supplemental table 4

Gene	Species	Method	Primer	Primer
TAMM41(P1)	Human	PCR	FP: TGCAGACGCTGCAGAGCTCGTGG	
TAMM41(P2)	Human	PCR	FP: CCAGAATAACTATGGCGCTGG	
TAMM41(P3)	Human	PCR	RP: GAGTCGTCCAGCAATGTATAAGT	
TAMM41(P4)	Human	PCR	FP: TGATCCCGACTGTGGAGATG	
TAMM41(P5)	Human	PCR	RP:GATGTTTTCCTCAGCCACCCTTTCCAC	
TAMM41(P6)	Human	PCR	FP:ACCTTCCGCAAGATCCTGTCTCA	
TAMM41(P7)	Human	PCR	RP:AGTGAACTATAAATCACTGACTTCTT	
TAMM41(P8)	Human	PCR	FP:GTGGAGATGTGGTGCGACTAG	
MT-CO2-FP	Human	QPCR	CAAACCTACGCCAAAATCCA	
MT-CO2-RP	Human	QPCR	GAAATGAATGAGCCTACAGA	
nDNA-GAPDH-FP	Human	QPCR	TGACAACAGCCTCAAGAT	
nDNA-GAPDH-RP	Human	QPCR	GAGTCCTTCCACGATACC	
DNM1L	Human	Clone	FP:ACGAATTCATGGAGGCGCTAATTCC	RP:ACCTCGAGTCACCAAAGATGAGTCT
PARK2	Zebrafish	Clone	FP:TTGGATCCATCGTTTTCGTGCGG	RP:GGAATTCCTCATTCGAACCAATGGTTTC
PARK2	Human	Clone	FP:CGGGATCCCGATAGTGTTTGTCAGG	RP:GGGAATTCCTACACGTCGAACCAG
TAMM41	Zebrafish	Clone	FP:GGAATTCATGAGTCTACCAGCTCTGC	RP:CCGCTCGAGTCATGCTGGTTTCCT
TAMM41	Human	Clone	FP:GGAATTCCATGGCGCTGCAGACGCTGC AGA	RP:CCGCTCGAGTCAGGATGTTTTCCTCAGCCAC
CDS1	Zebrafish	QPCR	FP: TGGCACACAAGTCAGGGAAC	RP:GCAGGCGGTAATGTTTCTTCA

PGS1	Zebrafish	QPCR	FP: TGGAAAAGACTTGTGTTCTCTGC	RP: GGGTGCTGAATCTGCCTCTG
FUNDC1	Zebrafish	QPCR	FP: CTTTCCATTCGCGTGCTGAT	RP: ATCCGGACACTCCACCCATC
MFN1B	Zebrafish	QPCR	FP: TCATTCAGGAGGTGTTGGCT	RP: CCTCATCTGTTCCCTCCACA
TFAM	Zebrafish	QPCR	FP: TAGCACTGCAACTCGAGGTC	RP: ATGGCTGCTTTTGTTCGGTG
NRF1	Zebrafish	QPCR	FP: CTCTTCAAGGCGGAGGACAG	RP: ATTCGAGTCGTTACGGGAGC
PGC1	Zebrafish	QPCR	FP: GCAATGAAGCATCCAACCTGT	RP: ATCACTGGCATTGGTCACGT
MFN2	Zebrafish	QPCR	FP: ACGGCATCTTTGAACAGCTG	RP: TCTTCGGGACAGGACTTCAC
PARK2	Zebrafish	QPCR	FP:ATGTGGTGCTGGTCTGCTC	RP:GATGCTGTGCTGGTtGTGTG
PINK1	Zebrafish	QPCR	FP:CATCGCTCACAGAGACCTCA	RP:CACTGCCATGGACACCTCAG
β-actin	Zebrafish	QPCR	FP:GATCTTCACTCCCCTTGTTCA	RP:GGCAGCGATTTCCTCATC
GAPDH	Human	QPCR	FP:GAAGGTGAAGGTCGGAGTC	RP:GAAGATGGTGATGGGATTTC
